# Classifying chronic pain using multidimensional pain-agnostic symptom assessments and clustering analysis

**DOI:** 10.1126/sciadv.abj0320

**Published:** 2021-09-08

**Authors:** Gadi Gilam, Eric M. Cramer, Kenneth A. Webber, Maisa S. Ziadni, Ming-Chih Kao, Sean C. Mackey

**Affiliations:** Division of Pain Medicine, Stanford University School of Medicine, Palo Alto, CA, USA.

## Abstract

Chronic pain conditions present in various forms, yet all feature symptomatic impairments in physical, mental, and social domains. Rather than assessing symptoms as manifestations of illness, we used them to develop a chronic pain classification system. A cohort of real-world treatment-seeking patients completed a multidimensional patient-reported registry as part of a routine initial evaluation in a multidisciplinary academic pain clinic. We applied hierarchical clustering on a training subset of 11,448 patients using nine pain-agnostic symptoms. We then validated a three-cluster solution reflecting a graded scale of severity across all symptoms and eight independent pain-specific measures in additional subsets of 3817 and 1273 patients. Negative affect–related factors were key determinants of cluster assignment. The smallest subset included follow-up assessments that were predicted by baseline cluster assignment. Findings provide a cost-effective classification system that promises to improve clinical care and alleviate suffering by providing putative markers for personalized diagnosis and prognosis.

## Teaser

Domain-general, patient-reported symptoms can robustly classify current severity of chronic pain and predict future pain levels.

## INTRODUCTION

Chronic pain is a global epidemic reflecting a health care crisis for the person suffering from it, their family, and society as a whole (*1*–*3*). More than 100 million individuals are affected by various chronic pain conditions in the United States alone, with medical expenses and lost productivity costing more than $635 billion annually and projected to become much worse (*4*–*6*). Primary chronic pain conditions present in various shapes and forms, commonly classified by anatomical location of experienced pain, from low-back pain and headaches to pelvic or bladder pain, including widespread nonspecific or overlapping pain (*7*, *8*). However, shared by all conditions is a global functional impairment that is manifested in the experience of multiple physical, “mental,” and social health symptoms, reflective of the biopsychosocial model of shared etiological factors across chronic pain conditions (*7*, *9*–*12*). While various studies aimed to uncover and classify subgroups of chronic pain (*13*–*22*), little is known whether a combination of domain-general symptoms agnostic to pain can be used to classify one’s chronic pain condition and subsequently serve as potential markers for clinical diagnosis and prognosis (*23*, *24*). A symptom-based approach may also reveal potentially modifiable factors as targets for therapeutic interventions. Therefore, we suggest a reversal of the common practice; instead of assessing patient-reported symptoms as features of the a priori determined pain condition, we examined whether such symptoms may serve to classify current and predict future pain condition. If confirmed, our approach could be used to support personalized and efficient treatment of individuals with chronic pain.

**Fig. 1.**
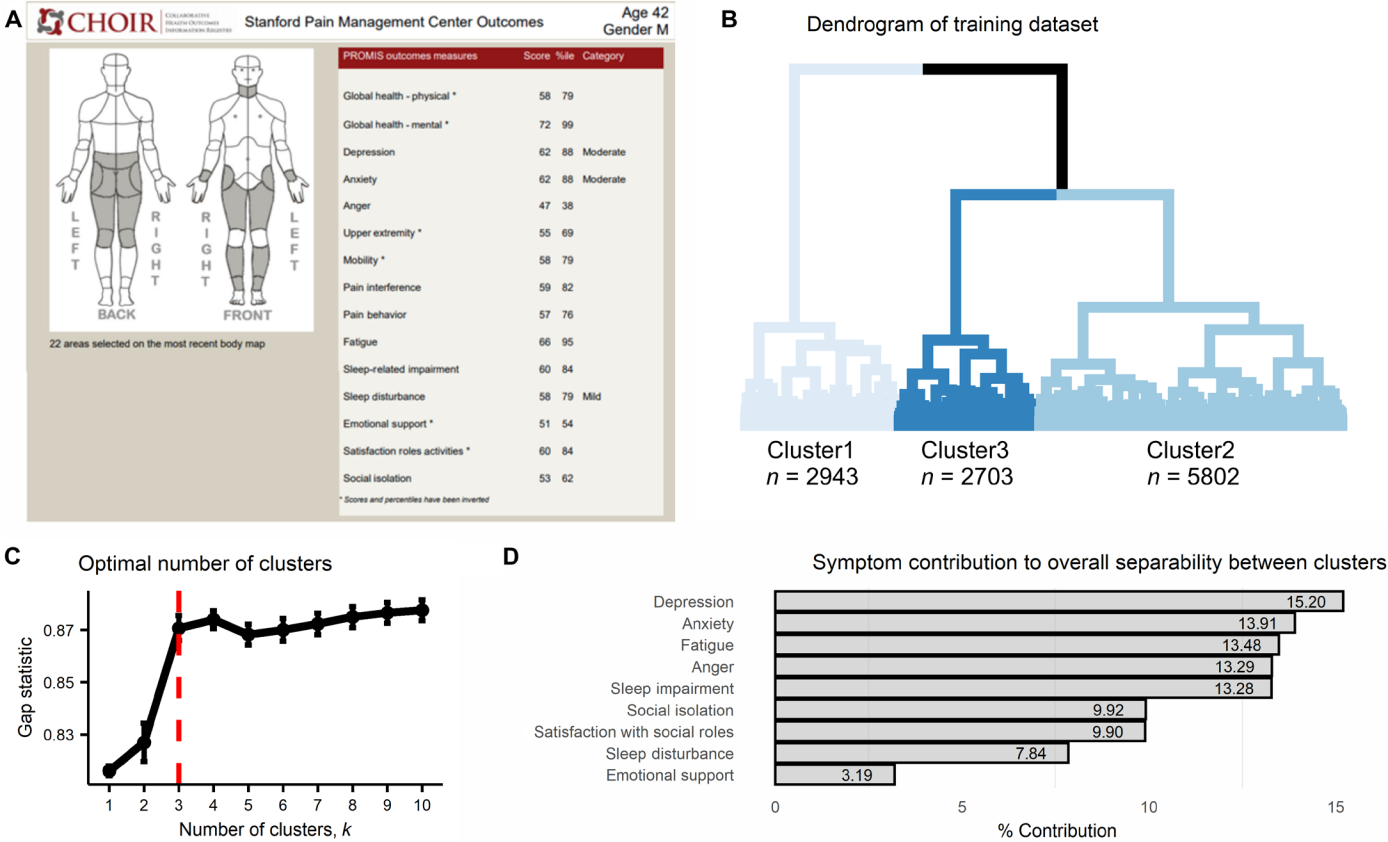
Cluster development. (**A**) An illustration of a simulated CHOIR report used at the Stanford Pain Clinics. The CHOIR body map with marked regions in pain is on the left, and multiple normalized symptom scores are listed on the right. (**B**) The dendrogram reflecting results of the agglomerative hierarchical clustering algorithm as implemented on the training dataset (*n* = 11448) and using the nine clustering symptoms. The three-cluster solution is reflected by the different shades of blue per cluster. Cluster1 composed of 25.71% of the patients (*n* = 2943), Cluster2 of 50.68% (*n* = 5802), and Cluster3 of 23.61% (*n* = 2703). (**C**) The plot shows the gap statistic values for different *k* number of clusters, and a red dashed line indicates the optimal solution of *k* = 3 since it is the smallest value of *k* that is within one standard deviation of the value of *k* that maximizes the gap statistic. The error bars represent one standard error of the estimated gap statistic. (**D**) The plot shows the percent contribution to the overall separability between clusters of each of the nine clustering symptoms, in the order of most contributing (depression = 15.20%) to least (emotional support = 3.19%).

We implemented unsupervised machine learning, specifically agglomerative hierarchical clustering analysis (AHCA) (*25*–*27*), on multidimensional patient-reported symptoms that assess physical, mental, and social health status factors, to identify idiosyncratic groups, or clusters of patients with chronic pain. Patients reflected a real-world clinical population with a heterogeneous mix of pain conditions seeking treatment at a tertiary academic pain clinic. As part of their routine initial evaluation, they completed multidimensional patient-reported assessments using Stanford’s Collaborative Health Outcomes Information Registry (CHOIR) registry-based learning health system (Fig. 1A) (*28*, *29*). We used nine symptoms for clustering based on the National Institutes of Health’s (NIH) Patient-Reported Outcomes Measurement Information System (PROMIS), which was designed and validated for precise and efficient measurement of health-related symptoms in patients with a wide variety of chronic health conditions (*30*). These symptoms were agnostic to nine pain-specific measures that we subsequently used to validate the diagnostic-like nature of the data-driven clusters independently.

Mechanistically, we aimed to uncover whether the multivariate pattern of symptoms and pain-specific measures characterizing each identified cluster reflects a general graded scale of severity or a differential pattern. Furthermore, given the centrality and comorbidity of mental health related factors with chronic pain, predominantly negative affect–related symptoms such as anxiety, depression, and anger (*10*, *31*, *32*), we expected these symptoms to be key drivers for the determination of cluster assignment, thus highlighting them as targets for treatment. We based cluster discovery on a training dataset of 11,448 patients and subsequently validated it in two additional datasets of 3817 and 1273 patients. The later dataset included follow-up assessments allowing us to examine whether cluster assignment at baseline would be predictive of pain-related measures at follow-up, thus providing potential prognostic-like validation of the identified clusters. Last, we examined the dynamics across assigned clusters between time points.

## RESULTS

### Demographic characteristics

Demographic characteristics of study participants are described in Table 1 (see also table S1).

**Table 1.** Participants’ demographic information as per dataset and across the three clusters. Number of patients is indicated, with % in parenthesis. * reflects the results of a Chi^2^ test (categories with less than a minimum of five patients per group were removed) comparing across clusters. Similar tests across datasets found no differences (*P* > 0.74).

	**Total**	**Cluster1**	**Cluster2**	**Cluster3**	P*
**Training dataset**					
**N (%)**	11,448 (69.22)	2943 (25.71)	5802 (50.68)	2703 (23.61)	
**Age (years)**					0.95
18–29	1398 (12.21)	372 (12.64)	660 (11.38)	366 (13.54)	
30–39	1966 (17.17)	510 (17.32)	954 (16.44)	502 (18.57)	
40–49	2127 (18.58)	506 (17.19)	1029 (17.74)	592 (21.90)	
50–59	2454 (21.44)	564 (19.16)	1264 (21.79)	626 (23.16)	
60–69	1974 (17.22)	543 (18.45)	1048 (18.06)	380 (14.06)	
≥70	1512 (13.21)	442 (15.02)	836 (14.41)	234 (8.66)	
No response	20 (0.17)	6 (0.2)	11 (0.19)	3 (0.11)	
**Sex**					0.95
Female	7340 (64.12)	1817 (61.74)	3700 (63.77)	1823 (67.44)	
Male	3723 (32.52)	1025 (34.83)	1904 (32.82)	794 (29.37)	
No response	385 (3.36)	101 (3.43)	198 (3.412)	86 (3.18)	
**Ethnicity**					0.98
Hispanic/Latino	1162 (10.15)	304 (10.33)	515 (8.88)	343 (12.69)	
Non-Hispanic/Non-Latino	8527 (74.48)	2230 (75.77)	4401 (75.85)	1896 (70.14)	
Patient refused	349 (3.05)	94 (3.19)	180 (3.1)	75 (2.77)	
Unknown	413 (3.61)	111 (3.77)	211 (3.64)	91 (3.37)	
No response	997 (8.71)	204 (6.93)	495 (8.53)	298 (11.02)	
**Race**					0.99
American Indian or Alaska Native	48 (0.42)	9 (0.31)	22 (0.38)	17 (0.63)	
Asian	935 (8.17)	287 (9.75)	469 (8.08)	179 (6.62)	
Asian, non-Hispanic	8 (0.07)	1 (0.03)	5 (0.09)	2 (0.07)	
Black or African American	405 (3.54)	102 (3.47)	184 (3.17)	119 (4.4)	
Black, non-Hispanic	8 (0.07)	3 (0.1)	2 (0.03)	3 (0.11)	
Native American, Hispanic	1 (0.01)	0 (0)	0 (0)	1 (0.04)	
Native American, non-Hispanic	1 (0.01)	0 (0)	1 (0.02)	0 (0)	
Native Hawaiian or other Pacific Islander	65 (0.57)	14 (0.48)	35 (0.6)	16 (0.59)	
Other	1921 (16.78)	487 (16.55)	929 (16.01)	505 (18.68)	
Other, Hispanic	11 (0.1)	1 (0.03)	9 (0.16)	1 (0.04)	
Other, non-Hispanic	8 (0.07)	2 (0.07)	5 (0.09)	1 (0.04)	
Patient refused	326 (2.85)	84 (2.85)	170 (2.93)	72 (2.66)	
Unknown	443 (3.87)	113 (3.84)	230 (3.96)	100 (3.7)	
White	6148 (53.7)	1598 (54.3)	3186 (54.91)	1364 (50.46)	
White, Hispanic	2 (0.02)	1 (0.03)	0 (0)	1 (0.04)	
White, non-Hispanic	106 (0.93)	30 (1.02)	54 (0.93)	22 (0.81)	
No response	1012 (8.84)	211 (7.17)	501 (8.63)	300 (11.1)	
**Marital status**					0.65
Married	5969 (52.14)	1809 (61.47)	3031 (52.24)	1129 (41.77)	
Separated	237 (2.07)	37 (1.26)	111 (1.91)	89 (3.29)	
Widowed	437 (3.82)	99 (3.36)	246 (4.24)	92 (3.4)	
Never married	2149 (18.77)	489 (16.61)	1065 (18.35)	595 (22.01)	
Living together	671 (5.86)	157 (5.33)	345 (5.95)	169 (6.25)	
Divorced	1218 (10.64)	229 (7.78)	623 (10.73)	366 (13.54)	
No response	767 (6.7)	123 (4.18)	381 (6.57)	263 (9.73)	
**Education (years)**					0.37
≤12	342 (2.99)	73 (2.48)	159 (2.74)	110 (4.07)	
13–16	3346 (29.23)	742 (25.21)	1606 (27.68)	998 (36.92)	
17–20	6033 (52.7)	1687 (57.32)	3135 (54.03)	1211 (44.80)	
≥21	1025 (8.95)	327 (11.11)	554 (9.55)	144 (5.33)	
No response	702 (6.13)	114 (3.87)	348 (6)	240 (8.88)	
**Validation dataset**					
**N (%)**	3817 (23.08)	931 (24.39)	2346 (61.46)	540 (14.15)	
**Age (years)**					0.76
18–29	490 (12.84)	119 (12.78)	299 (12.75)	72 (13.33)	
30–39	644 (16.87)	156 (16.76)	386 (16.45)	102 (18.89)	
40–49	713 (18.68)	144 (15.47)	439 (18.71)	130 (24.07)	
50–59	768 (20.12)	165 (17.72)	485 (20.67)	118 (21.85)	
60–69	682 (17.81)	198 (21.27)	414 (17.65)	70 (12.96)	
≥70	510 (13.36)	147 (15.79)	316 (13.47)	47 (8.7)	
No response	10 (0.26)	2 (0.21)	7 (0.3)	1 (0.19)	
**Sex**					0.87
Female	2496 (65.39)	589 (63.27)	1564 (66.67)	343 (63.52)	
Male	1193 (31.25)	322 (34.59)	698 (29.75)	173 (32.04)	
No response	128 (3.35)	20 (2.15)	84 (3.58)	24 (4.44)	
**Ethnicity**					0.87
Hispanic/Latino	386 (10.11)	94 (10.1)	221 (9.42)	71 (13.15)	
Non-Hispanic/ Non-Latino	2797 (73.28)	688 (73.9)	1747 (74.47)	362 (67.04)	
Patient refused	122 (3.2)	36 (3.87)	68 (2.9)	18 (3.33)	
Unknown	151 (3.96)	47 (5.05)	87 (3.71)	17 (3.15)	
No response	361 (9.46)	66 (7.09)	223 (9.51)	72 (13.33)	
**Race**					0.99
American Indian or Alaska Native	21 (0.55)	5 (0.54)	12 (0.51)	4 (0.74)	
Asian	323 (8.46)	90 (9.67)	189 (8.06)	44 (8.15)	
Asian, non-Hispanic	2 (0.05)	0 (0)	1 (0.04)	1 (0.19)	
Black or African American	124 (3.25)	32 (3.44)	71 (3.03)	21 (3.89)	
Black, non-Hispanic	2 (0.05)	1 (0.11)	1 (0.04)	0 (0)	
Native American, Hispanic	0 (0)	0 (0)	0 (0)	0 (0)	
Native American, non-Hispanic	1 (0.03)	1 (0.11)	0 (0)	0 (0)	
Native Hawaiian or other Pacific Islander	15 (0.39)	4 (0.43)	8 (0.34)	3 (0.56)	
Other	647 (16.95)	161 (17.29)	388 (16.54)	98 (18.15)	
Other, Hispanic	5 (0.13)	2 (0.21)	3 (0.13)	0 (0)	
Other, non-Hispanic	0 (0)	0 (0)	0 (0)	0 (0)	
Patient refused	121 (3.17)	37 (3.97)	66 (2.81)	18 (3.33)	
Unknown	134 (3.51)	42 (4.51)	77 (3.28)	15 (2.78)	
White	2021 (52.95)	480 (51.56)	1283 (54.69)	258 (47.78)	
White, Hispanic	0 (0)	0 (0)	0 (0)	0 (0)	
White, non-Hispanic	36 (0.94)	9 (0.97)	22 (0.94)	5 (0.93)	
No response	365 (9.56)	67 (7.2)	225 (9.59)	73 (13.52)	
**Marital status**					0.76
Married	1991 (52.16)	529 (56.82)	1230 (52.43)	232 (42.96)	
Separated	99 (2.59)	15 (1.61)	59 (2.51)	25 (4.63)	
Widowed	157 (4.11)	49 (5.26)	89 (3.79)	19 (3.52)	
Never married	684 (17.92)	162 (17.40)	420 (17.90)	102 (18.89)	
Living together	243 (6.37)	55 (5.91)	152 (6.48)	36 (6.67)	
Divorced	396 (10.37)	81 (8.7)	250 (10.66)	65 (12.04)	
No response	247 (6.48)	40 (4.3)	146 (6.22)	61 (11.3)	
**Education (years):**					0.21
≤12	108 (2.83)	25 (2.69)	53 (2.26)	30 (5.56)	
13–16	1140 (29.87)	241 (25.89)	700 (29.84)	199 (36.85)	
17–20	1995 (52.27)	529 (56.82)	1245 (53.07)	221 (40.93)	
≥21	331 (8.67)	93 (9.99)	207 (8.82)	31 (5.74)	
No response	243 (6.37)	43 (4.62)	141 (6.01)	59 (10.93)	
**Longitudinal dataset** **(at baseline)**					
**N (%)**	1273 (7.70)	263 (20.66)	827 (64.96)	183 (14.38)	
**Age (years)**					0.14
18–29	169 (13.28)	34 (12.93)	111 (13.42)	24 (13.11)	
30–39	184 (14.45)	35 (13.31)	123 (14.87)	26 (14.21)	
40–49	263 (20.66)	42 (15.97)	172 (20.8)	49 (26.78)	
50–59	275 (21.6)	47 (17.87)	181 (21.89)	47 (25.68)	
60–69	229 (17.99)	56 (21.29)	144 (17.41)	29 (15.85)	
≥70	151 (11.86)	49 (18.63)	95 (11.49)	7 (3.83)	
No response	2 (0.16)	0 (0)	1 (0.12)	1 (0.55)	
**Sex**					0.80
Female	865 (67.95)	172 (65.4)	560 (67.71)	133 (72.68)	
Male	367 (28.83)	84 (31.94)	239 (28.9)	44 (24.04)	
No response	41 (3.22)	7 (2.66)	28 (3.39)	6 (3.28)	
**Ethnicity**					0.89
Hispanic/Latino	129 (10.13)	31 (11.79)	71 (8.59)	27 (14.75)	
Non-Hispanic/ Non-Latino	998 (78.4)	207 (78.7)	652 (78.84)	139 (75.96)	
Patient refused	43 (3.38)	7 (2.66)	30 (3.63)	6 (3.28)	
Unknown	47 (3.69)	10 (3.8)	33 (3.99)	4 (2.19)	
No response	56 (4.4)	8 (3.04)	41 (4.96)	7 (3.83)	
**Race**					0.95
American Indian or Alaska Native	10 (0.79)	3 (1.14)	6 (0.73)	1 (0.55)	
Asian	90 (7.07)	20 (7.6)	56 (6.77)	14 (7.65)	
Asian, non-Hispanic	0 (0)	0 (0)	0 (0)	0 (0)	
Black or African American	31 (2.44)	7 (2.66)	15 (1.81)	9 (4.92)	
Black, non-Hispanic	0 (0)	0 (0)	0 (0)	0 (0)	
Native American, Hispanic	0 (0)	0 (0)	0 (0)	0 (0)	
Native American, non-Hispanic	0 (0)	0 (0)	0 (0)	0 (0)	
Native Hawaiian or other Pacific Islander	3 (0.24)	1 (0.38)	1 (0.12)	1 (0.55)	
Other	239 (18.77)	58 (22.05)	136 (16.44)	45 (24.59)	
Other, Hispanic	0 (0)	0 (0)	0 (0)	0 (0)	
Other, non-Hispanic	0 (0)	0 (0)	0 (0)	0 (0)	
Patient refused	35 (2.75)	7 (2.66)	22 (2.66)	6 (3.28)	
Unknown	51 (4.01)	8 (3.04)	35 (4.23)	8 (4.37)	
White	740 (58.13)	148 (56.27)	501 (60.58)	91 (49.73)	
White, Hispanic	1 (0.08)	0 (0)	1 (0.12)	0 (0)	
White, non-Hispanic	12 (0.94)	2 (0.760)	10 (1.21)	0 (0)	
No response	61 (4.79)	9 (3.42)	44 (5.32)	8 (4.37)	
**Marital status**					0.52
Married	688 (54.05)	151 (57.41)	461 (55.74)	76 (41.53)	
Separated	19 (1.49)	2 (0.76)	12 (1.45)	5 (2.73)	
Widowed	42 (3.3)	14 (3.32)	22 (2.66)	6 (3.28)	
Never married	272 (21.37)	49 (18.63)	174 (21.04)	49 (26.78)	
Living together	80 (6.28)	18 (6.84)	49 (5.93)	13 (7.1)	
Divorced	157 (12.33)	27 (10.27)	100 (12.09)	30 (16.39)	
No response	15 (1.18)	2 (0.76)	9 (1.09)	4 (2.19)	
**Education (years)**					0.06
≤12	21 (1.65)	4 (1.52)	12 (1.45)	5 (2.73)	
13–16	384 (30.16)	72 (27.37)	231 (27.93)	81 (44.26)	
17–20	747 (58.68)	159 (60.46)	502 (60.70)	86 (46.99)	
≥21	109 (8.56)	23 (8.75)	77 (9.31)	9 (4.92)	
No response	12 (0.94)	5 (1.9)	5 (0.6)	2 (1.09)	

### Cluster discovery, characterization, reliability, and validity

The dendrogram reflecting results of the AHCA as implemented on the training dataset is shown in Fig. 1B. On the basis of the gap statistic, we grouped patients into an optimal number of three clusters (Fig. 1C). In line with our expectation, the negative affect–related clustering symptoms of depression, anxiety, and anger were the most important factors driving the clustering process, ranked first, second, and fourth, respectively, and contributing 42.4% to the overall separability between clusters (Fig. 1D).

**Fig. 2.**
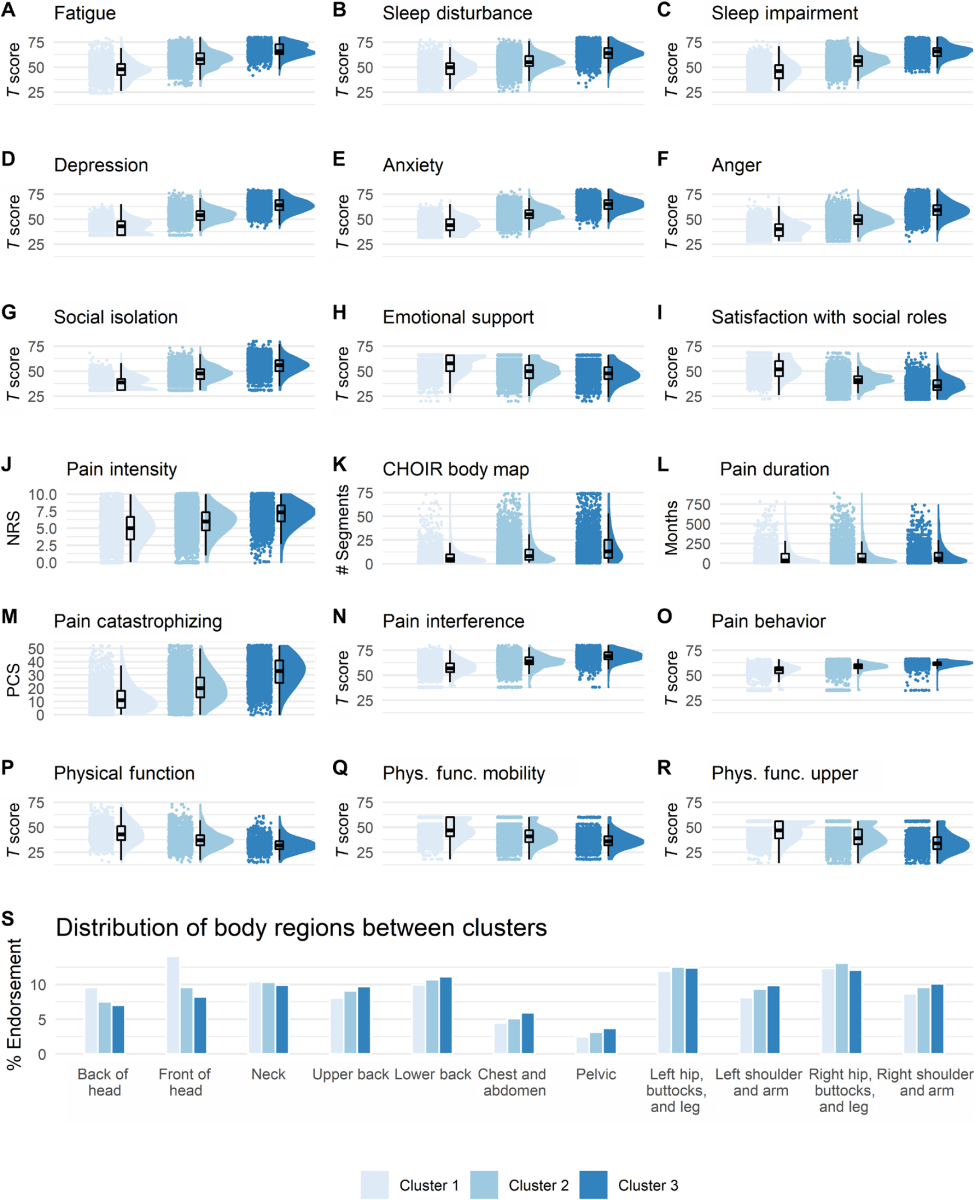
Cluster characterization and diagnostic-like validation. A graded scale of severity is manifested across all clustering symptoms (**A** to **I**) and all pain-specific measures (**J** to **R**), such that Cluster1 reflects a low severity, Cluster2 reflects a medium severity, and Cluster3 reflects the worst severity. Raincloud plots combining jittered raw data, data distribution, and boxplots were generated using open source code (*93*). Complementary descriptive and inferential statistical information is provided in Table 2. (**S**) The plot shows the % endorsement of 11 body regions as distributed in each of the clusters. There was no significant association in the distribution of % endorsed body regions between the clusters (*P* = 0.99). NRS, Numerical Rating Scale; PCS, Pain Catastrophizing Scale.

We labeled the clusters Cluster1, Cluster2, and Cluster3 to reflect the graded scale of severity that characterized all clustering symptoms (Table 2 and Fig. 2, A to I) and all pain-specific measures (Table 2 and Fig. 2, J to R). These results provided initial validation of these clusters such that Cluster1 reflects the least severe condition, Cluster3 reflects the worst, and Cluster2 is in between, with substantial effect sizes across all comparisons (Table 2). Since PROMIS instruments are normed to the general U.S. population, we could inform that Cluster1 was on average 0.60 SD better than the norm in the clustering symptoms but 0.46 SD worse than the norm in the subset of PROMIS-based pain-specific measures. Cluster2 was 0.36 SD and 1.05 SD, and Cluster3 was 1.20 SD and 1.54 SD, all worse than the norm in the clustering symptoms and pain-specific measures.

**Table 2.** Clustering symptoms and pain-specific measures as per the three clusters in the training dataset. M, mean; C1, cluster1; C2, cluster2; C3, cluster3.

	**Descriptive (M ± SD)**			**Main effect of cluster**			**C1 versus C2**		**C1 versus C3**		**C2 versus C3**	
	**C1**	**C2**	**C3**	**F**	**df**	P*	***P* ^†^**	**Cohen’s D**	***P* ^†^**	**Cohen’s D**	***P* ^†^**	**Cohen’s D**
**Clustering symptoms:**												
Fatigue	47.36 ± 8.82	57.97 ± 7.63	67.64 ± 6.68	4849.83	2, 11,445	<0.0001	<0.0001	1.29	<0.0001	2.59	<0.0001	1.35
Sleep disturbance	48.84 ± 8.92	55.26 ± 7.35	64.24 ± 7.58	2742.75	2, 11,445	<0.0001	<0.0001	0.79	<0.0001	1.86	<0.0001	1.20
Sleep impairment	45.78 ± 9.09	55.45 ± 6.79	65.92 ± 6.55	5218.07	2, 11,445	<0.0001	<0.0001	1.21	<0.0001	2.54	<0.0001	1.57
Depression	42.29 ± 6.82	53.87 ± 6.34	63.82 ± 6.95	7515.97	2, 11,445	<0.0001	<0.0001	1.76	<0.0001	3.13	<0.0001	1.50
Anxiety	44.2 ± 7.26	54.76 ± 6.83	64.81 ± 6.56	6334.6	2, 11,445	<0.0001	<0.0001	1.50	<0.0001	2.98	<0.0001	1.50
Anger	39.11 ± 7.71	49.18 ± 7.38	59.26 ± 8.23	4860.57	2, 11,445	<0.0001	<0.0001	1.33	<0.0001	2.53	<0.0001	1.29
Social isolation	38.22 ± 6.67	47.48 ± 7.29	55.61 ± 7.89	4034.82	2, 11,445	<0.0001	<0.0001	1.33	<0.0001	2.38	<0.0001	1.07
Emotional support	56.94 ± 9.23	49.99 ± 8.56	47.4 ± 8.81	933.58	2, 11,445	<0.0001	<0.0001	0.78	<0.0001	1.06	<0.0001	0.30
Satisfaction with soc. roles	52.91 ± 9.14	41.71 ± 7.1	35.79 ± 7.6	3614.51	2, 11,445	<0.0001	<0.0001	1.37	<0.0001	2.04	<0.0001	0.80
**Pain-specific measures**												
Pain intensity	4.93 ± 2.34	5.86 ± 1.98	7.01 ± 1.76	734.67	2, 11,445	<0.0001	<0.0001	0.50	<0.0001	1.49	<0.0001	0.92
Number of bodymap segments	7.62 ± 8.43	11.39 ± 11.39	17.99 ± 16.09	461.19	2, 9,916	<0.0001	<0.0001	0.27	<0.0001	1.29	<0.0001	0.58
Pain duration	86.5 ± 119.99	95.91 ± 122.92	101.8 ± 116.73	9.74	2, 9,477	<0.0001	<0.002	0.08	<0.0001	0.18	0.061	0.07
Pain interference	57.34 ± 7.97	63.52 ± 6.45	69.29 ± 5.78	2227.08	2, 11,445	<0.0001	<0.0001	1.01	<0.0001	2.62	<0.0001	1.41
Pain behavior	54.26 ± 6.82	58.39 ± 4.39	61.53 ± 3.14	1575.07	2, 11,445	<0.0001	<0.0001	1.08	<0.0001	2.34	<0.0001	1.41
Physical function	44.13 ± 10.18	37.4 ± 8.49	32.5 ± 6.95	467.67	2, 4,081	<0.0001	<0.0001	0.87	<0.0001	1.94	<0.0001	1.00
Phys. func. mobility	47.69 ± 9.88	41.49 ± 9.26	36.79 ± 8.13	642.56	2, 7,361	<0.0001	<0.0001	0.71	<0.0001	1.66	<0.0001	0.82
Phys. func.-up. extrem.	46.64 ± 9.52	40.49 ± 10.3	34.71 ± 9.77	640.15	2, 7,361	<0.0001	<0.0001	0.61	<0.0001	1.64	<0.0001	0.84
Pain catastrophizing	12.67 ± 9.88	20.99 ± 10.97	32.37 ± 11.15	2237.26	2, 10,795	<0.0001	<0.0001	0.75	<0.0001	2.54	<0.0001	1.44

*Bonferroni threshold for ANOVA main effects of cluster is set at *P* = 0.0028.

†Bonferroni threshold for t-test comparisons between each two clusters is set at *P* = 0. 0009.

Although the pattern of severity also manifested in the number of self-reported body segments in pain, with Cluster3 indicative of potential widespread and/or overlapping chronic pain conditions, we found no significant associations between specific body regions (table S2 and fig. S1) and any of the clusters (Chi^2^ = 3.23, *P* = 0.99; Fig. 2S). Cluster1 was only descriptively associated with more pain in the front of the head (14.07%) compared to Cluster2 (9.61%) and Cluster3 (8.22%; Chi^2^ = 1.75, *P* = 0.41). Similarly, none of the demographic characteristics were significantly associated with any specific cluster (Table 1). We replicated the same pattern of results across clustering symptoms and pain-specific measures in the validation (table S3 and fig. S2) and the longitudinal datasets (table S4 and fig. S3), except for pain duration, for which we found no differences between the three clusters (*P* > 0.12). This supported the reliability and validity of the identified clusters. However, two critical questions arose that we addressed in the following two sections.

### Can we generate idiosyncratic groups of patients using only pain intensity?

**Fig. 3.**
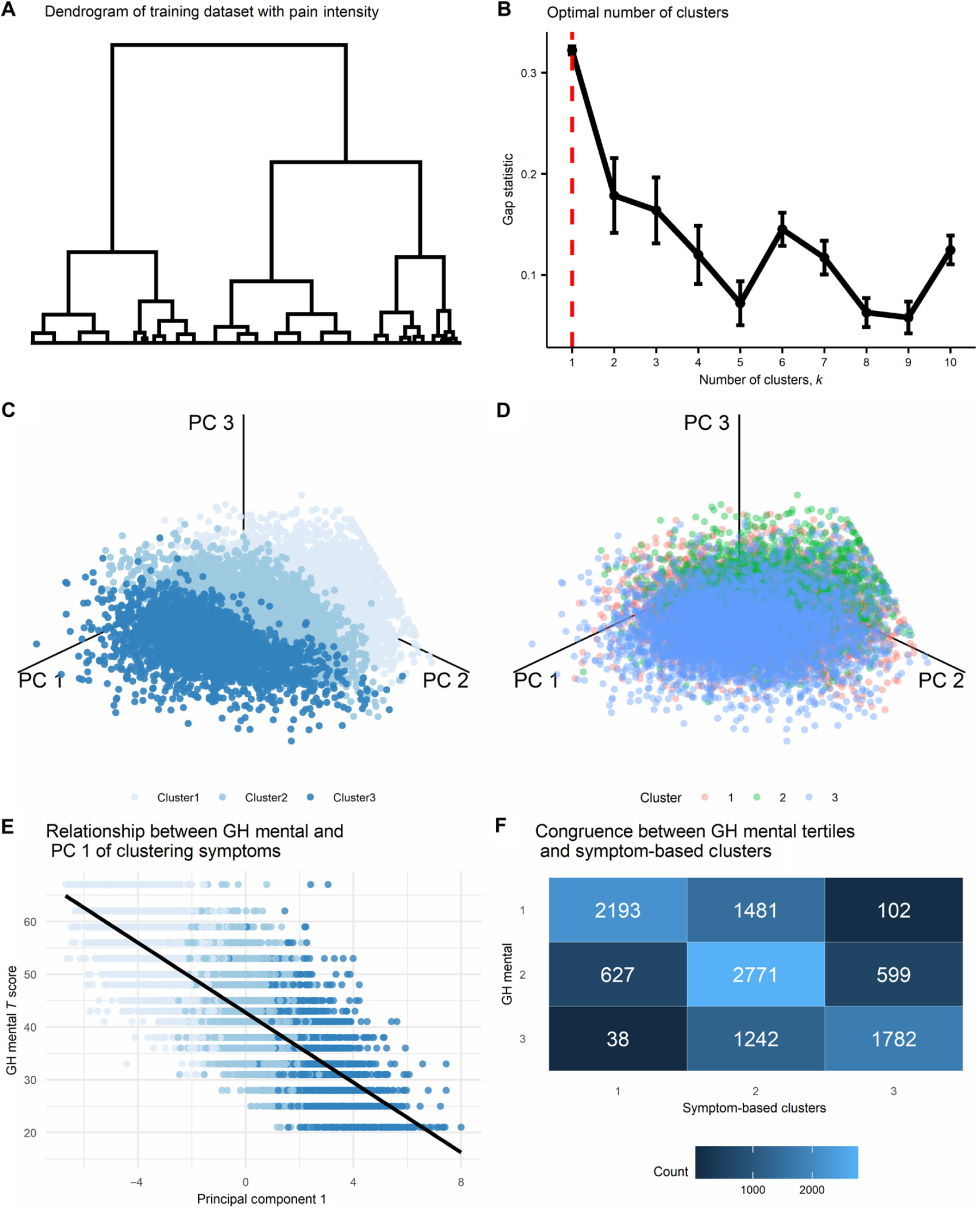
Alternatives to the symptom-based clustering solution. (**A**) The resulting dendrogram reflecting results of the agglomerative hierarchical clustering algorithm (AHCA) as implemented on the training dataset (*n* = 11448) and using the pain intensity measure. The tree is not clustered since the optimal solution was of one cluster. (**B**) The plot shows the gap statistic values for different *k* number of clusters, and a red dashed line indicates the optimal solution of *k* = 1. The error bars represent one standard error of the estimated gap statistic. (**C**) and (**D**) Plots that show the distribution of all data points in the training dataset on the three primary principal components (PCs) derived from the nine-dimensional clustering symptoms and colored according to either the three clusters generated from the AHCA of these symptoms (C) or according to the three clusters generated from the AHCA of pain intensity (D). The separability between clusters is clearly seen in the clustering symptoms’ solution, while a substantial overlap is seen in the pain intensity solution. (**E**) The plot shows the correlation between the first PC and the PROMIS global health (GH) mental subscale in the training dataset (available for *n* = 10,835): *r* = −0.78, *P* < 0.001. (**F**) Congruence matrix between the clustering symptoms’ three-cluster solution, and the tertiles that were labeled according to the PROMIS GH mental subscale. The overall level of congruence was 62.26%.

The clustering solution identified three clusters portraying a graded scale of severity across all clustering symptoms and all pain-specific measures. Pain intensity is a common measure for assessing one aspect of the severity of pain and thus can be used to examine whether one such variable can similarly obtain a solution that generates idiosyncratic groups of patients. Although AHCA is commonly applied on multiple measurements, it technically requires one variable at minimum, and thus, we applied AHCA on the training dataset using only pain intensity. The dendrogram reflecting the results of the AHCA is shown in Fig. 3A. The gap statistic indicated an optimal number of one cluster (Fig. 3B). We nevertheless selected the nonoptimal three-cluster solution to compare it with the clustering symptoms-based solution directly. To visualize this comparison, we applied principal components analysis (PCA) (*26*, *27*) on the nine-dimensional clustering symptoms (see fig. S4A for scree plot and fig. S4B for clustering symptoms’ contribution to the first three principal components). To evaluate the separability between the clusters of the two solutions, we plotted the entire training dataset using the first three principal components and colored the data points based on the three clusters of the clustering symptoms solution (Fig. 3C) and of the pain intensity solution (Fig. 3D). The separability between clusters is clearly seen in the clustering symptoms solution, while a substantial overlap is seen in the pain intensity solution, indicating that using pain intensity alone cannot capture a similar solution.

### To what degree does the clustering solution reflect a latent mental health–related construct?

As we initially expected, the negative affect- or mental health–related clustering symptoms were the most important factors driving the clustering process and contributing most to the first principal component (42.2% of the explained variance; fig. S4B). To estimate the degree by which the underlying data structure of the clustering symptoms reflect a mental health–related construct, we calculated the Pearson correlation coefficient between the first three principal components derived by the above mentioned PCA and the PROMIS Global Health Mental subscale. To note, this measure was available for *n* = 10,835 of the training dataset. The first principal component explained 55.42% of the variance in the data structure (fig. S4A) and had a correlation of *r* = −0.78 with the PROMIS Global Health Mental subscale (Fig. 3E). The correlation with the second and third principal components, which explained 12.28 and 8.83% of the variance in the data structure (fig. S4), were *r* = 0.11 and *r* = −0.02, respectively. As expected, this reconfirms mental health as a key construct in the data’s underlying structure but not the only.

To further illustrate this point, we split the range of possible PROMIS Global Health Mental scores into tertiles and labeled them in order of severity (1, 2, and 3) to match the clustering symptoms’ cluster solution labeling. We then quantified the level of congruence between these two sets of labels by counting how many patients were assigned by each of the solutions to the same cluster label and how many were mismatched between the clusters (Fig. 3F). The level of congruence was 76.73, 50.44, and 71.77% for Cluster1, Cluster2, and Cluster3, respectively, and with an overall 62.26% congruence. Together, it is clear that mental health is a primary component in the data’s underlying structure. Still, the proposed clustering solution reflects more than a latent mental health–related construct, particularly at the intermediate Cluster2.

### Predictive validation and cluster dynamics over time

**Fig. 4.**
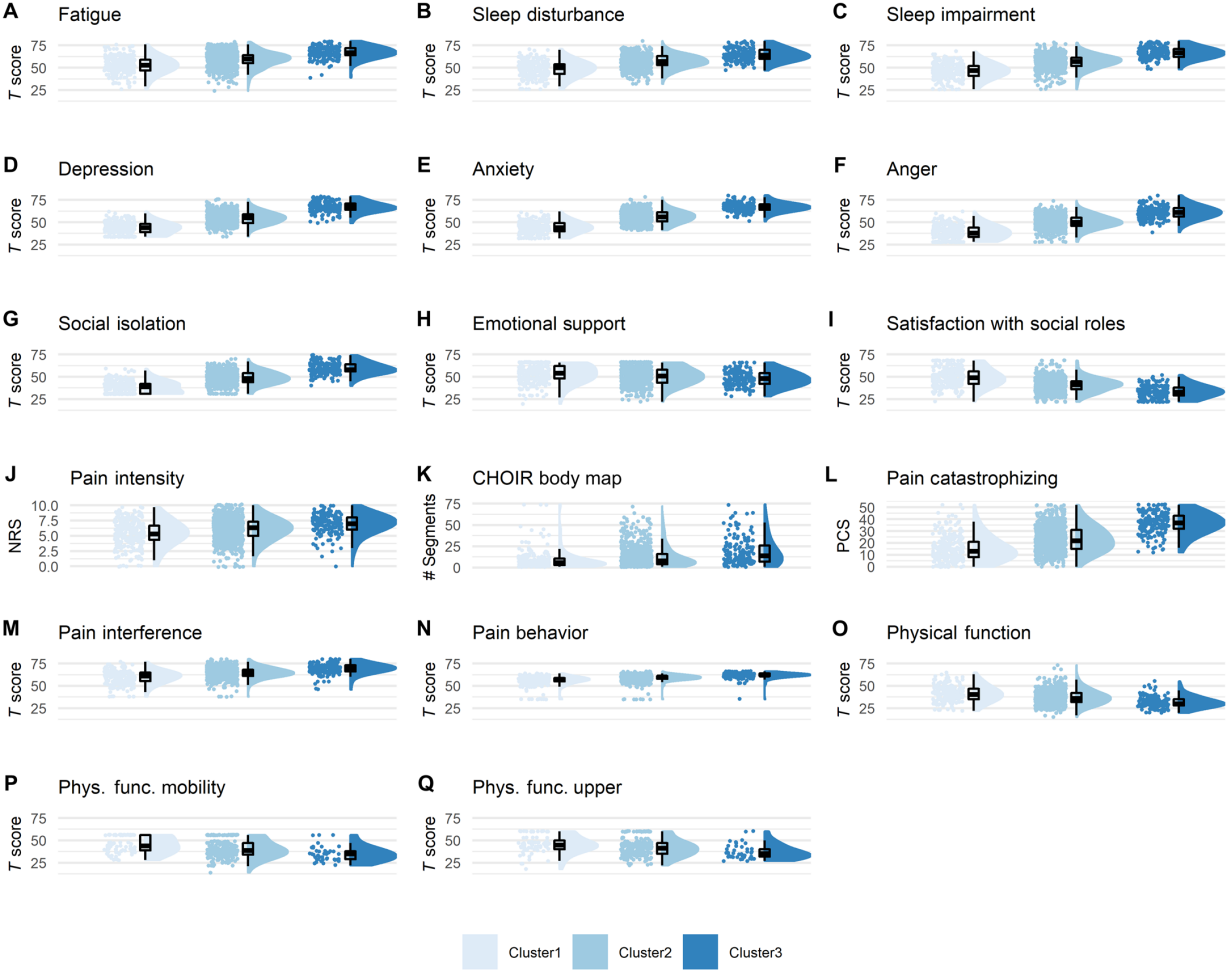
Predictive validation of the clusters. All raincloud plots for the clustering symptoms (**A** to **I**) and for the pain-specific measures (**J** to **Q**) reflect severity of assessment at follow-up (longitudinal dataset, *n* = 1273), based on cluster identification at baseline. Complementary descriptive and inferential statistical information is provided in Table 3. The graded scale of severity is manifested also here, such that those labeled as Cluster1 at baseline continue to have at the group level the lowest level of severity across all measures, and the same for Cluster2 and Cluster3 being the medium and worst severity, respectively.

**Fig. 5.**
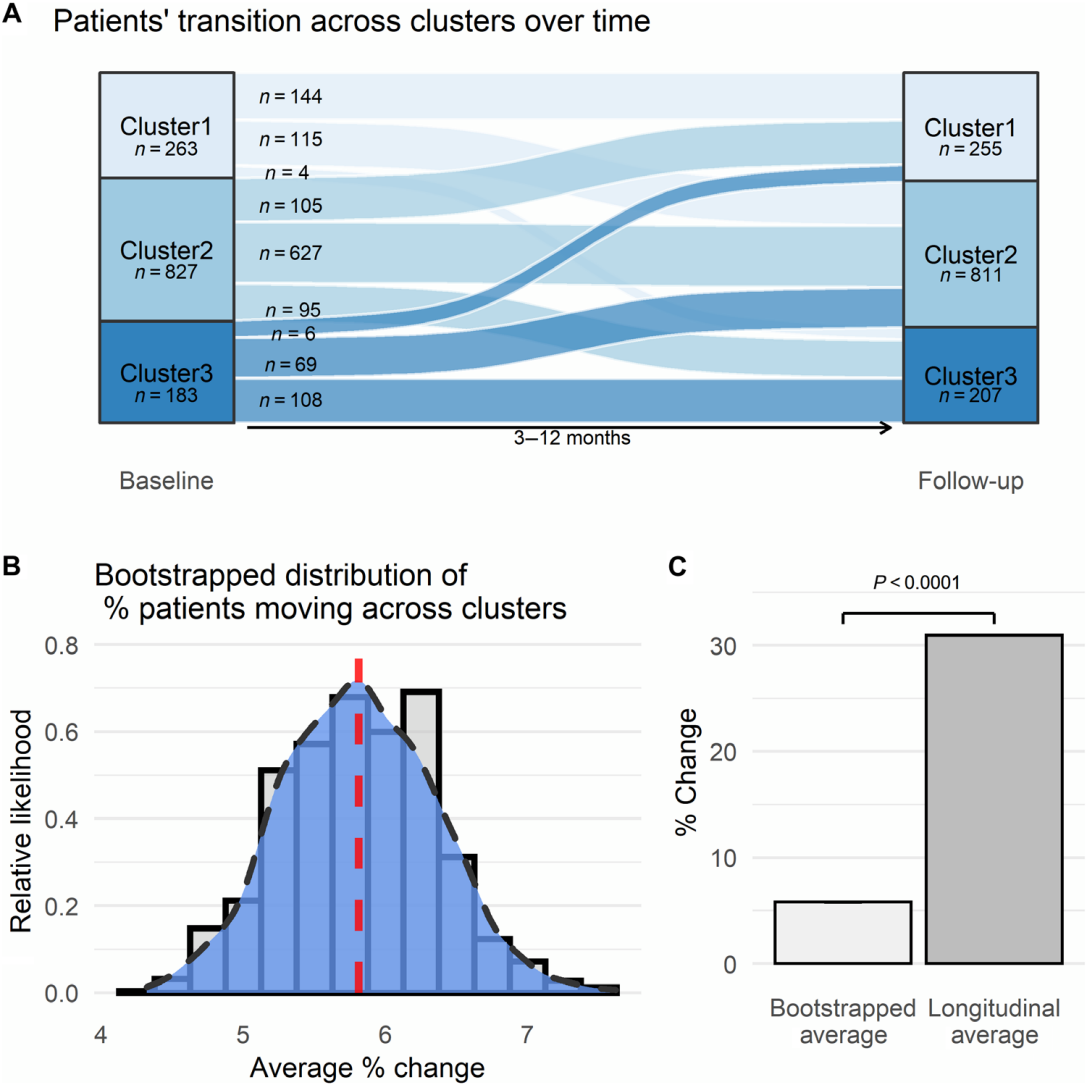
Cluster dynamics over time. (**A**) Sankey plot indicating the transition of patients across clusters over time in the longitudinal dataset (*n* = 1273). Width of lines reflect the extent of movement between time points. One hundred and eighty patients (14.14%) had an improvement in their condition, 214 patients (16.81%) had a worsening in their condition, and 879 (69.05%) remained in the same cluster, between baseline assessment and follow-up. (**B**) Plot of the smoothed kernel density estimate, or relative likelihood, of observing a % change in the bootstrapped distribution of patients moving across clusters within a randomized range of ±3-point measurement error. The red dashed line marks the average set at *M* = 5.81% ± 0.54SD. The smoothed curve indicates the exact likelihood of observing an exact percent change (i.e., the *x*-axis value). The bars behind the density curve reflect the same information, averaged at 0.2 sized bins. (**C**) Bar plot comparing the average percent change of patients moving across clusters from the bootstrap distribution (SE = 0.017, too small to be seen), with the actual 30.95% found over time in the longitudinal dataset.

After controlling for the time between the two assessments (3 to 12 months), we were able to demonstrate substantial differences between clusters as identified at baseline in all clustering symptoms (Table 3 and Fig. 4, A to I) and pain-specific measures at follow-up (Table 3 and Fig. 4, J to Q). Cluster1 continued to reflect the least severe condition, Cluster3 the worst, and Cluster2 in between, again with substantial effect sizes across all comparisons (Table 3). These results validate the prognostic-like nature of the clusters and suggest that the graded scale of severity remains consistent at follow-up at the group level. Nevertheless, cluster identification at follow-up demonstrates that while most patients (*n* = 879, 69.05%) remained within their same cluster between the two time points, there were movements across clusters (Fig. 5A): 180 patients (14.14%) had an improvement in their condition and moved from Cluster3 to Cluster2 (*n* = 69, 5.42%) or Cluster1 (*n* = 6, 0.47%) and from Cluster2 to Cluster1 (*n* = 105, 8.25%); and 214 patients (16.81%) had a worsening in their condition and moved from Cluster1 to Cluster2 (*n* = 115, 9.03%) or Cluster3 (*n* = 4, 0.31%) and from Cluster2 to Cluster3 (*n* = 95, 7.46%). We compared the total movement of patients across clusters between time points (*n* = 394, 30.95%) to a bootstrapped distribution of patients moving across clusters within potential measurement error [mean (*M*) = 5.81% ± 0.54 SD; Fig. 5B], which indicated a significant amount of movement (*t*_(df = 999)_ = 1477.18, *P* < 0.0001; Fig. 5C). This suggests that the changes across clusters are meaningful, potentially indicating an interaction between treatment effects and regression to the mean (*33*). Thus, cluster assignment is not a static condition; rather, various factors might affect the long-term dynamics across clusters, offering a window of opportunity for personalized interventions.

**Table 3.** Clustering symptoms and pain-specific measures in the longitudinal dataset at follow-up, as per the three clusters assigned at baseline.

	**Descriptive (M ± SD)**			**Main effect of cluster**			**C1 versus C2**		**C1 versus C3**		**C2 versus C3**	
	**C1**	**C2**	**C3**	**F**	**df**	P*	***P* ^†^**	**Cohen’s D**	***P* ^†^**	**Cohen’s D**	***P* ^†^**	**Cohen’s D**
**Clustering symptoms:**												
Fatigue	53.49 ± 10.23	59.53 ± 9.33	65.75 ± 9.02	61.27	3, 1269	<0.0001	<0.0001	0.62	<0.0001	1.27	<0.0001	0.68
Sleep disturbance	50.5 ± 9.42	56.35 ± 8.62	62.76 ± 9.12	69.52	3, 1269	<0.0001	<0.0001	0.65	<0.0001	1.32	<0.0001	0.72
Sleep impairment	49.68 ± 9.76	57.25 ± 8.46	64.46 ± 8.72	104.78	3, 1269	<0.0001	<0.0001	0.83	<0.0001	1.60	<0.0001	0.84
Depression	46.19 ± 8.70	54.6 ± 8.35	63.13 ± 8.39	149.00	3, 1269	<0.0001	<0.0001	0.99	<0.0001	1.98	<0.0001	1.02
Anxiety	47.35 ± 9.04	56.33 ± 8.24	64.5 ± 8.45	153.95	3, 1269	<0.0001	<0.0001	1.04	<0.0001	1.96	<0.0001	0.98
Anger	42.04 ± 9.74	50.36 ± 9.14	59.05 ± 9.57	121.55	3, 1269	<0.0001	<0.0001	0.88	<0.0001	1.76	<0.0001	0.93
Social isolation	40.68 ± 8.25	48.54 ± 8.49	57.9 ± 8.66	149.69	3, 1269	<0.0001	<0.0001	0.94	<0.0001	2.04	<0.0001	1.09
Emotional support	54.92 ± 9.64	51.66 ± 9.38	47.99 ± 8.44	20.93	3, 1269	<0.0001	<0.0001	0.34	<0.0001	0.76	<0.0001	0.41
Satisfaction with soc. roles	48.37 ± 10.11	42.65 ± 8.22	36.14 ± 7.51	75.65	3, 1269	<0.0001	<0.0001	0.62	<0.0001	1.37	<0.0001	0.83
**Pain-specific measures:**												
Pain intensity	5.12 ± 2.31	5.60 ± 2.05	6.39 ± 1.93	13.76	3, 1269	<0.0001	<0.005	0.24	<0.0001	0.88	<0.0001	0.58
Number of bodymap segments	8.87 ± 10.84	12.44 ± 12.58	17.41 ± 15.45	14.62	3, 1088	<0.0001	<0.0005	0.25	<0.0001	0.96	<0.0001	0.45
Pain interference	60.72 ± 8.29	63.63 ± 7.31	67.91 ± 7.07	33.25	3, 1269	<0.0001	<0.0001	0.40	<0.0001	1.39	<0.0001	0.86
Pain behavior	56.23 ± 5.57	58.69 ± 4.18	61.07 ± 2.65	46.04	3, 1269	<0.0001	<0.0001	0.70	<0.0001	1.64	<0.0001	1.27
Physical function	40.26 ± 9.90	36.86 ± 8.39	31.96 ± 6.75	12.89	3, 488	<0.0001	<0.005	0.45	<0.0001	1.40	<0.0001	1.03
Phys. func. mobility	43.86 ± 10.51	40.04 ± 10.09	35.13 ± 9.48	18.63	3, 777	<0.0001	<0.0001	0.39	<0.0001	1.22	<0.0001	0.73
Phys. func.-up. extrem.	44.92 ± 10.00	41.69 ± 8.70	37.88 ± 8.56	14.52	3, 777	<0.0001	<0.0001	0.37	<0.0001	1.14	<0.0001	0.63
Pain catastrophizing	12.57 ± 10.99	18.72 ± 12.14	29.57 ± 12.62	73.07	3, 1269	<0.0001	<0.0001	0.50	<0.0001	1.98	<0.0001	1.22

*Bonferroni threshold for ANCOVA main effects of cluster is set at *P* = 0.0029.

†Bonferroni threshold for *t* test comparisons between each two clusters is set at *P* = 0.001.

To provide an estimate of what entails a change in cluster assignment, we calculated the average of absolute change across the nine clustering symptoms’ scores between the two time points, as well as the average number of symptoms that had an absolute change beyond the estimated measurement error. We compared these values between the group of *n* = 394 patients that moved across clusters and the *n* = 879 patients that remained in the same cluster. The patients moving across clusters between time points had a larger absolute change in symptoms’ scores (7.59 ± 3.65) and a larger number of symptoms that changed beyond the measurement error (6.00 ± 1.79) compared to those patients remaining in the same cluster [5.18 ± 2.15, *t*(1271) = 14.72, *P* < 0.0001; and 4.96 ± 1.74, *t*(1271) = 9.78, *P* < 0.0001, respectively].

## DISCUSSION

In our study, we offer a novel biopsychosocial-inspired approach to classify patients with chronic pain, resulting in the identification of three robust idiosyncratic groups of patients and generating putative markers that can classify current and predict future severity of chronic pain in a graded manner, regardless of their formal diagnosis or their underlying etiology. We applied a data-driven clustering algorithm on multidimensional self-reported symptom assessments that are agnostic to pain. These assessments were collected through CHOIR, Stanford’s registry-based learning health care system (*34*), and belonging to more than 16,000 real-world patients seeking treatment at a tertiary academic pain clinic. The assessments can be completed using an electronic device from almost any place, in about 15 min, and with hardly any need for assistance from staff. These findings can be instrumental in supporting treatment selection and pain management in a personalized health care platform, especially in the current forward-triage approach to health care in which a clinician might not be able to physically examine a patient (*35*). Moreover, findings inspire further research into the biological and behavioral mechanisms that characterize the identified clusters.

The three identified groups reflect a graded scale of severity. They are therefore labeled Cluster1, Cluster2, and Cluster3, with higher numbers indicative of a more severe condition, as shown in all assessments, including those used for clustering and those specific for pain, except for pain duration since onset of chronic pain. The strongest drivers separating between clusters are the negative affect–related factors. No apparent demographic factors significantly differ between clusters. The overall group characteristics initially found on a subset of more than 11,000 patients reliably reproduced in two additional subsets consisting of about 5000 more patients. Moreover, one of these subsets comprising 1273 patients included follow-up assessments, the severity of which were predicted on the basis of the baseline cluster assignment. Examining the dynamics across clusters between baseline and follow-up assessments indicated that cluster assignment is not a static condition, suggesting that various factors might affect improvement or worsening of the pain condition. Thus, beyond the diagnostic- and prognostic-like nature of these symptom-based putative markers, future clinical and research efforts should examine whether and to what extent they will indicate response to various treatments (*23*, *24*). This will be of importance to further determine the extent by which changes in cluster assignment reflect “real” change rather than potential measurement errors or other statistical phenomena.

A primary concern of chronic pain health care is identifying safe and effective treatments tailored to the patient’s particular needs. Evidence-based approaches have been called to address this challenge by generating classification systems that focus on the fine-grained multidimensional and mechanistic substrates of chronic pain conditions (*36*, *37*). However, translating these systems into clinically interpretable and applicable tools is challenging, especially if these systems require costly and burdensome medical tests (*38*). Consequently, there have been growing efforts to generate empirical classifications of patients with chronic pain based on relatively simple assessments that may advance our understanding of the underlying substrates of chronic pain and potentially inform and support clinical decision-making (*13*–*22*). These efforts differ in sample sizes (from approximately a hundred to thousands), type of chronic pain groups (heterogeneous, specific diagnoses, or even pain-free), type of measures (subjective and/or objective), type of analytic approach (e.g., supervised versus unsupervised algorithms), and the number of resulting clusters (mostly in the range of two to four groups), among other. While a detailed review of the various classifications approaches goes beyond the scope of the current work, two such solutions are noteworthy.

One of the first efforts to empirically classify patients with chronic pain (*22*) identified three clusters based on the multidimensional pain inventory (MPI) (*39*). The MPI assesses psychosocial and behavioral factors related to the experience of chronic pain, such as pain severity and interference, affective distress, social support, and behavioral activities. The cluster labeled as dysfunctional had relatively high levels of pain and emotional distress, low levels of perceived life control and behavioral activation, and intermediate levels on various social related factors. The interpersonally distressed cluster had intermediate levels on most factors but was low on social support–related factors. The minimizer/adaptive copers cluster had low levels of pain and emotional distress, high levels of perceived life control and behavioral activation, and high social support. The subgroups initially developed using patients with heterogeneous chronic pain, later reproduced in other chronic pain diagnostic groups, such as low back pain, headache, and patients with temporomandibular disorder (TMD) (*40*). Mixed effects were found for the potential association between MPI-based classification and treatment outcomes (*41*–*44*).

A more recent effort (*14*) used numerous clinical characteristics, psychosocial questionnaires, and measures of autonomic function and multimodal pain sensory testing in patients with TMD and TMD-free controls from several locations in the United States to identify three groups based on best possible characterization of chronic TMD. An adaptive cluster consisting mostly of the controls had better autonomic function, the lowest sensitivity to pain, and the lowest levels of various psychosocial characteristics and symptoms. A global symptoms cluster, half of which were TMD cases, had high levels of sensitivity to pain and of psychosocial characteristics and symptoms. Follow-up analysis indicated that TMD-free controls from this group had greater risk of developing first-onset TMD. An additional pain-sensitive cluster, a quarter of which were TMD cases, had intermediate levels of psychosocial characteristics and symptoms, coupled with heightened sensitivity to experimental pain. An algorithm based on a much smaller subset of the initial measurements, including muscle pain sensitivity, somatization, anxiety, and depression, reproduced and generalized the clusters in additional cohorts from different locations, including patients with chronic overlapping pain conditions and clinical patients most commonly diagnosed with TMD, fibromyalgia, trigeminal neuralgia, and headache (*16*).

In most clinical settings, as in research, chronic pain is predominantly diagnosed by the relevant anatomical location of pain (*7*, *8*). The symptom-based classification system proposed here, similarly to the clustering efforts just described, was inspired by the biopsychosocial approach (*9*–*12*). We aimed to expand the common practice by integrating evidence-based and patient-centric information that go beyond the potential underlying objective location and manifestation of pathological disease and incorporate the subjective and personal experience and expression of the illness. However, unlike previous clustering efforts, we do so by focusing on domain-general patient-reported symptoms that are agnostic to pain and thus commonly considered secondary in classifying patients with chronic pain rather than a potential starting point. Unlike the dominant diagnostic system, we find no associations between specific locations of experienced pain and identified cluster. Nevertheless, the number of body regions in pain increased with severity, indicating that patients with widespread and/or overlapping chronic pain conditions suffer more than those with a more localized pain condition. In line with previous clustering approaches and other research (*14*, *45*), findings thus evidence a diminished reliance on specific anatomical locations of experienced pain when assessing and classifying the severity of impairment in primary pain conditions and potentially when considering treatment avenues. Together, although there are similarities and differences between the current and previous clustering approaches, it is clear that additional research is required to further corroborate the symptom-based classification system proposed here. Moreover, to maximize utility, future efforts should aim to integrate between data-driven clustering approaches as our own and those described above to provide the idiosyncratic complexity of pain within the currently used diagnostic systems.

As expected, negative affect–related symptoms emerged as key factors driving the clustering process. Researchers have previously demonstrated similar negative affect–related metrics to be central in clustering patients with chronic pain (*13*, *14*). As we further confirmed, a global measure of mental health was a key construct in the data’s underlying structure. This reverberates with the crucial role of mental health in chronic pain (*10*, *31*) and as a potential underlying pathological mechanism differing between clusters. Moreover, there is currently an ongoing paradigm shift in psychology and psychiatry, calling for the classification of psychopathology as a hierarchy of continuous dimensions rather than describing it through discrete diagnostic categories (*46*). On top of the hierarchy is a global factor termed the “p factor,” generally ranging from low to high psychopathological severity and cutting through all psychopathological disorders to account for their nonspecific and overlapping manifestation of symptoms (*47*, *48*). The resemblance to chronic pain is astounding. The empirical findings presented here suggest that pain as a field of study and treatment should consider establishing a similar hierarchy of continuous global transdiagnostic dimensions to improve the ability to address the challenges of chronic pain. Moreover, this echoes our contemporary perspective on the need for more synergistic interactions between the research and clinical fields of pain and mental health, specifically regarding the centrality of affective components to these fields (*31*).

Our findings confirm existing notions of a general graded scale of severity of chronic pain illness (*45*, *49*, *50*). However, our approach extends previous efforts in terms of the combination of scale, scope, computational approach, and especially in that we use multidimensional domain-general symptoms that are agnostic to pain. This is advantageous for two main reasons. First, it may highlight potentially modifiable targets for intervention. As we anticipated, negative affect–related factors, namely depression, anxiety, and anger, were key drivers in cluster assignment at the group level. Fortunately, there is a flourishing of treatment strategies aimed to reduce negative affect–related symptomatology (*51*–*56*). Moreover, findings indicate that the distribution of symptoms severity and of patients across clusters is, to a certain extent, blended (e.g., Figs. 2, A to R, and 3C). This suggests that a health care clinician may consider the particular pattern of symptoms at the individual patient level regarding the assigned cluster and use this information to guide and support clinical decision-making contextually. For example, we may envision a patient assigned to the lower severity Cluster1 but has relatively high levels of sleep dysfunction that a clinician could address with specific sleep-related treatments (*57*).

A second advantage of the domain-general symptoms approach is that it may be implemented and potentially generalizable to other chronic illnesses requiring symptom management beyond the specific pathophysiology of their disease, like cancer, immune disorders, and cardiovascular diseases among many others. Notably, the graded classification of severity resonates with other illnesses that are characterized by a staged progression of disease, such as cancer (*58*), heart (*59*) and kidney diseases (*60*), diabetes (*61*), and more. Here, however, we did not use objective and etiological-based metrics, and future integration of genetic, metabolic, inflammatory, and/or anatomical and functional neuroimaging metrics can substantially improve our understanding of the biological mechanisms underlying the identified symptom-based clusters and potentially lead to improved (bio)marker properties (*23*, *24*).

The U.S. National Pain Strategy has drawn attention to a subgroup of patients with chronic pain—those with persistent high-impact chronic pain. These patients suffer from the most severe and debilitating illness, substantially restricting and interfering with daily life activities, and requiring increased health care expenditure (*3*, *62*, *63*). The prevalence of high-impact chronic pain is estimated to be between 5 and 15% of the adult U.S. population (10 to 30 million people) (*3*, *5*, *63*). Compared to lower but still clinically significant chronic pain, high-impact chronic pain was associated with unfavorable health outcomes, limitations in daily activity, negative coping strategies, elevated distress, increased health care costs, and higher usage and dosage of opioid medication (*50*, *64*). With the potential collateral personal, societal, and financial impact of long-term opioid medication (*65*), it is particularly crucial to better identify and understand people suffering from and at increased risk of high-impact chronic pain. Within our proposed classification system, Cluster3 may be reflective of such a group of patients: An overall most severe condition characterizes it, manifested at the group level by highest levels of pain interference, widespread and/or overlapping chronic pain conditions, low levels of physical function, fatigue, depression, and basically in every measure that we assessed. Early identification of these patients is essential for the provision of more comprehensive and costly pain assessments (e.g., psychological, medical, etc.) that better inform treatment approaches (e.g., physical or psychosocial therapy, medical interventions, etc.). Future research efforts may contrast current conceptualizations of high-impact chronic pain with the characteristics of Cluster3 and consider to what extent it supports this early identification.

There are notable limitations to our study. While our cohort is large, supporting generalizability of the sample and stability of the discovered clusters, it is still restricted to the San Francisco Bay Area and the outlining Northern California region and potentially also to patients who can afford specialized medical treatment in a tertiary academic clinic. Future efforts will need to generalize our findings to other locations with different demographic, sociocultural, and economic characteristics. In this regard, there are known demographic disparities related to pain health care (*66*, *67*) that were not captured by the identified clusters. This may be attributed to the particular characteristics of the cohort (table S3), for example, being primarily White (53.87%) and with above college level of education (61.92%). However, there are some descriptive trends worth noting. Across datasets (table S3), Cluster3 was generally characterized by younger age, lower education level, more females, more patients identifying as Hispanic or Latino, less of them identifying as White, and less reporting being married. To note, the recent classification solution described above (*14*, *16*) similarly indicated more females, but in contrast found older age, both to be associated with the non-adaptive clusters. The range of ages differed between studies, with about 50% of the current sample older than 50 while the cohorts used by the previous clustering effort were mostly below 44. Although it is crucial to better understand the nature of such associations, it is essential to highlight that we can only address most of these factors through a necessary systematic change in health care.

In addition, in terms of cohort characteristics, we had no reliable data on formal diagnostic codes within our cohort. Although findings indicate no association between the identified clusters and anatomical pain location, which is commonly used to support formal definitions of chronic pain conditions, future efforts should examine the relationship between formal diagnoses of disease state and cluster membership. Notably, previous CHOIR studies were able to obtain and indicate a multitude of formal diagnoses (*28*, *29*)—including neuropathic, thoracolumbar, orofacial, visceral, and various musculoskeletal pain conditions, as well as fibromyalgia and complex regional pain syndrome, among many others—and these can be assumed to be part of the current cohort. Thus, our findings seem to be generalizable at least to varying types of chronic pain conditions.

The symptoms used for clustering are based on NIH’s PROMIS system, which has potential limitations, since although the symptoms were validated for their psychometric properties (*30*, *68*–*73*), they are still based on subjective self-reports and thus prone to potential biases and demand characteristics. Other studies using various clustering approaches have incorporated more objective measurements, with a better characterization of their underlying mechanistic substrates. Most have used various multimodal pain sensory testing that map on to various nociceptive pathways (*14*, *15*, *17*, *18*, *20*, *21*). While incorporating objective measures with better understanding of their underlying pathophysiology is a clear next step for this research, using the PROMIS system offers substantial advantages. PROMIS-based *T* scores are normed to the general U.S. population and thus easily comparative across cohorts. PROMIS is also an inexpensive and easily administered system, using short forms or computerized adaptive testing (CAT) to reduce time and patient burdens, and is already in wide usage in many settings, even beyond chronic pain, thus allowing others to take a similar approach as ours or to engage with our freely available cluster classifier (https://git.io/Jn8m1) for additional utilization in clinical and research settings. Moreover, previous findings show associations between various PROMIS measures and potential biomarkers in both pain and nonpain clinical contexts (*74*–*76*). Last, the clusters differ in pain-specific measure that are non-PROMIS based, such as pain intensity and pain catastrophizing, and this solidifies the validity and generalizability beyond PROMIS-based measures.

In conclusion, our symptom-based approach and findings offer significant diagnostic- and prognostic-like utility for a cost-effective, graded severity classification system of patients with chronic pain, potentially generalizable to other chronic illnesses. Our study’s exploratory nature requires further research to reconfirm and generalize the identified clusters in different chronic pain cohorts, as well as experimental and mechanistic studies to uncover their etiological basis. Nevertheless, this system promises to support clinical decision-making, affecting the day-to-day functioning of patients with chronic pain, and encourages investigations into new treatment opportunities oriented toward a precision- and evidence-based approach to relieve the burdens of people suffering from chronic illness and improve their quality of life. It thus reflects a synergy between theory-driven scientific research, clinical care, and technological advancement that aims to facilitate personalized health care by closing on the bedside-to-bench-to-bedside loop.

## MATERIALS AND METHODS

### General data acquisition procedures and dataset definition

Data were collected using Stanford University’s CHOIR (http://choir.stanford.edu), a registry-based, learning health care system that administers an electronic survey assessing self-reported demographic information, pain characteristics, and multiple domains of health status in real-world clinical settings (Fig. 1A) (*34*). Patients presenting for consultation at Stanford Pain Management Center locations throughout the San Francisco Bay Area and broader Northern California region, USA, with the main site located in Redwood City, complete the survey as part of their routine clinical care. While intended for completion at home using personal computers or hand-held devices, patients may complete the survey before their appointment at clinic check-in using a tablet computer. Survey completion is encouraged, yet optional, and is based on patients’ willingness and ability to collaborate. Patients may therefore choose not to respond to certain items or assessments. These procedures were approved by the Stanford University School of Medicine Institutional Review Board (IRB). Informed consent was waived by the IRB, as CHOIR data were collected for clinical care and quality improvement purposes.

Data analyzed were from a retrospective review of all collected surveys since CHOIR’s inception in October 2013 through August 2019. Our initial data extraction included 24,389 records, from which we removed records based on the following criteria: noncompleted or test records (6002), missing data in any of the nine measures used for clustering (as detailed below; 1651), duplicated records (136), and age below 18 years (62). From the resulting 16,538 surveys belonging to 16,538 different patients, we extracted a longitudinal dataset of 1273 patients with a follow-up survey between 3 and 12 months later and again with a minimal requirement of having complete data for the nine assessments used for clustering at both time points. We chose this time frame since 3 months is considered the minimal threshold for diagnosing primary chronic pain (*7*). The upper threshold of 12 months allowed to keep a substantially large proportion of the dataset for cluster discovery validation. The resulting 15,265 patients were randomly split on the basis of a 75%:25% allocation into a training dataset of 11,448 patients used for cluster discovery and an additional validation dataset of 3817 patients.

### Measures

#### *Demographic characteristics*

Demographic characteristics included age, sex, ethnicity, race, marital status, and years of education.

### Clustering symptoms

The nine symptoms assessing health-related functionality and used as the basis for the clustering procedures were from the NIH’s PROMIS (*30*, *68*–*70*, *72*, *73*). We divided these nine symptoms into three domains: (i) the physical domain (fatigue, sleep disturbance, and sleep impairment), (ii) the mental or negative affect domain (depression, anxiety, and anger), and (iii) the social domain (social isolation, emotional support, and satisfaction with social roles and activities). Response items are contextualized to the frequency of the experienced symptom in the past 7 days (e.g., “in the past seven days how often did you feel tired?” and “in the past seven days I felt worthless”), and responses were marked on a 1 to 5 scale (1 = never, 5 = always). Each measured symptom was completed using CAT, based on item response theory–derived metrics. CAT reduces the time needed to complete each measured symptom because patients respond only to a subset of items from the relevant PROMIS item bank. This subset of items is selected by the CAT algorithm to have the most information to precisely characterize the symptom for the patient, with a minimum of 4 (for adults) and a maximum of 12 items, although typically not more than eight items, taking a minute or two per measured symptom (*30*, *71*). The full range of total items responded by patients for the nine clustering symptoms was therefore between 36 and 108 items. Typically, completing all nine symptom measurements should take about 15 min (*77*, *78*).

Ultimately, a standardized *T* score for each PROMIS symptom is generated for each patient. A score of 50 reflects the mean of the U.S. general population, with an SD of 10. Higher *T* scores reflect more of the measures’ symptom. We further extracted data of a PROMIS-based global health measure, specifically the Global Health Mental subscale that consisted of four items assessing general mental health, quality of life, satisfaction with social activities, and emotional problems (*79*). While for most measures such as fatigue or depression, higher *T* scores indicated a worse condition, for emotional support, satisfaction with social roles, global health mental, and physical function (see below), higher *T* scores reflected a better condition. Further details regarding measure development and validation are available at www.healthmeasures.net.

### Pain-specific measures

Pain-specific measures were used independently of the clustering process to validate the diagnostic-like nature of the data-driven generated clusters in terms of pain-related constructs. A composite score of pain intensity was calculated by averaging three self-reported pain intensity measures. These measures used a common and validated (*80*) 11-point numeric rating scale of 0 to 10 (0 = no pain, 10 = pain as bad as you can imagine) for worst and average pain in the last 7 days and current pain. The number of body segments in which chronic pain is experienced was self-reported by patients, who were asked to mark locations of pain on a reliable and valid CHOIR body map scheme that included 36 anterior and 38 posterior symmetrical body segments for a maximum total of 74 segments (Fig. 1A) (*81*). This measure was used to reflect the extent of pain throughout the body. A group of physicians recoded these 74 body segments into 11 body regions (table S1 and fig. S1) subsequently used to examine specific locations in which patients experienced pain. There are 6 body segments in the male and female versions of the CHOIR body map that are labeled with different codes. Patients who do not report their gender are given the female version of the body map by default. These differences were re-coded to match for the correct body region across all participants. Pain duration was calculated as the number of months from onset of chronic pain that was self-reported by patients.

Additional measures assessed using PROMIS instrumentation included pain interference with daily life activities, pain behavior, and physical function (*30*, *70*). In September 2016 and moving forward, physical function was assessed using two separate measures in CHOIR, reflecting physical function of the upper extremity and lower mobility (*82*). Across the entire dataset, most patients had the two separate measures (61.32%). We analyzed each of the three physical function measures separately to be able to differentiate between them.

The last pain-specific measure was pain catastrophizing, reflecting maladaptive cognitions such as rumination, magnification, and helplessness, in response to actual or anticipated pain. Pain catastrophizing has been associated with poor outcomes, maintenance, and worsening of chronic pain illness (*83*–*85*). We used the Pain Catastrophizing Scale that previously demonstrated sound psychometric properties (*86*–*88*) to measure the frequency with which a patient engages in catastrophic thought patterns and consists of 13 self-reported items on a 0 to 4 scale (0 = not at all, 10 = all the time).

### Statistical analysis

Programming and analyses were conducted using a combination of R version 4.0.0 (*89*), RStudio version 1.2.5042 (*90*), and IBM SPSS version 26. Relevant open source R codes are available at https://git.io/Jn8m1.

### Cluster discovery

Hierarchical clustering is a well-established unsupervised machine learning technique that aims to discover groups or clusters of observations within a dataset without needing to a priori determine the specific characteristics of each cluster (*25*–*27*). Observations within the same cluster are expected to have similar characteristics, while different clusters are expected to have dissimilar characteristics. A cluster-tree diagram or dendrogram is a mathematical and pictorial representation of a cluster solution.

We implemented an AHCA on the training dataset and using the nine clustering symptoms, to assign each patient to a cluster. AHCA implements an iterative process in which the two most similar observations (i.e., patients or groups of patients) are fused to form a superordinate cluster until all observations belong to one single cluster. Two parameters important for this process are a distance metric that determines how similar observations are to each other and a linkage method to fuse similar observations. The agglomerative coefficient can then assess how tightly packed each cluster is within a cluster solution. We used the Euclidian distance metric combined with the Ward linkage method as it optimized the agglomerative coefficient compared to four other linkage methods (table S5).

We subsequently used the gap statistic to determine the optimal number of clusters, *k* (*91*). The gap statistic compares the within-cluster sum of squares of a certain k-clusters solution to the expected within-cluster sum of squares under a null distribution with no clusters. An ideal solution will have a small within-cluster sum of squares and therefore a large gap statistic. We calculated the gap statistic for *k* between 1 and 10. The smallest value of *k* that is within 1 SD of the value of *k* that maximizes the gap statistic should be chosen as the optimal number of clusters.

We next aimed to determine the relative importance of each clustering symptom to the clustering process, i.e., to the separability between clusters. We computed the cluster centroid (*25*, *27*), which is the average value of each clustering symptom for all of the observations in that cluster, and then calculated the total Euclidian distance between all cluster centroids. The average amount each clustering symptom contributed to the distance between each clusters’ centroid, divided by the total sum of all clustering symptoms’ contribution to the total Euclidean distance between all cluster centroids, provides a percent contribution to the overall separability between clusters.

### Cluster characterization, reliability, and validity

Univariate analysis of variance (ANOVA) and subsequent *t* tests were used to examine differences in clustering symptoms and in pain-specific measures between the identified clusters, with Bonferroni correction applied to account for multiple comparisons. Chi^2^ tests were used to examine the differential distribution of demographic factors and of body regions between the clusters and determine whether specific body regions were associated with any of the identified clusters. This sequence of tests was conducted initially on the training dataset to assess the clusters’ diagnostic-like potential and subsequently on the validation and the baseline of the longitudinal datasets to assess the reliability of cluster assignment and validate the cluster’s characteristics in other sets of patients. A nearest centroid classifier (*25*, *27*) was generated to assign or label a cluster to a “new” patient, based on the shortest Euclidian distance between the values of the clustering symptoms of that patient and each clusters’ centroids.

### Predictive validation and cluster dynamics over time

Univariate analyses as described above were used to examine differences in clustering symptoms and in pain-specific measures between clusters as assigned at baseline, using data from the follow-up. To control for time-related effects, we added the number of days between the two assessments as covariate. This provided prognostic-like validation of the clusters.

Next, the nearest centroid classifier was implemented on the follow-up dataset to assess patient movement across clusters between the baseline and follow-up time points. Also, we used a bootstrap procedure (*26*, *27*) to assess whether patients’ movement across clusters over time was due to potential error in measurement of the clustering symptoms or potentially to the clusters’ ability to portray real improved or worsening of their condition. Since the PROMIS CAT engine uses a stopping criterion such that the standard error of the *T* score drops below a specified level of 3.0 *T* score metric points (*92*), we randomly jittered the original *T* score for each patient and for each of the clustering symptoms at baseline, within that error threshold, i.e., ± 3.0 *T* score points. The nearest centroid classifier was implemented on each patient’s simulated data to assign a cluster. We then assessed movement across clusters between the simulated dataset and the follow-up dataset and calculated the number and subsequently the percent of patients moving across clusters. This procedure was repeated 1000 times to generate a bootstrapped distribution of the percent of patients moving across clusters within measurement error. This distribution allowed us to calculate the probability of the actual percent of patients moving across clusters between the baseline and follow-up time points being attributed to measurement error.

## Notes

View/request a protocol for this paper from *Bio-protocol*.
