# Supplementary material for: Classifying chronic pain using multidimensional pain-agnostic symptom assessments and clustering analysis
Source: Sci Adv. 2021 Sep 8;7(37):eabj0320. doi: 10.1126/sciadv.abj0320 (PMC8442889; doi:10.1126/sciadv.abj0320)
Supplement: Supplementary file 1 — Figs. S1 to S4 Tables S1 to S5 [file sciadv.abj0320_sm.pdf]

Science Advances
AAAS

Supplementary Materials for

Classifying chronic pain using multidimensional pain-agnostic symptom assessments and clustering analysis

Gadi Gilam*, Eric M. Cramer, Kenneth A. Webber II, Maisa S. Ziadni, Ming-Chih Kao, Sean C. Mackey

*Corresponding author. Email: gadi.gilam@stanford.edu

Published 8 September 2021, *Sci. Adv.* **7**, eabj0320 (2021)
DOI: 10.1126/sciadv.abj0320

This PDF file includes:

Figs. S1 to S4
Tables S1 to S5

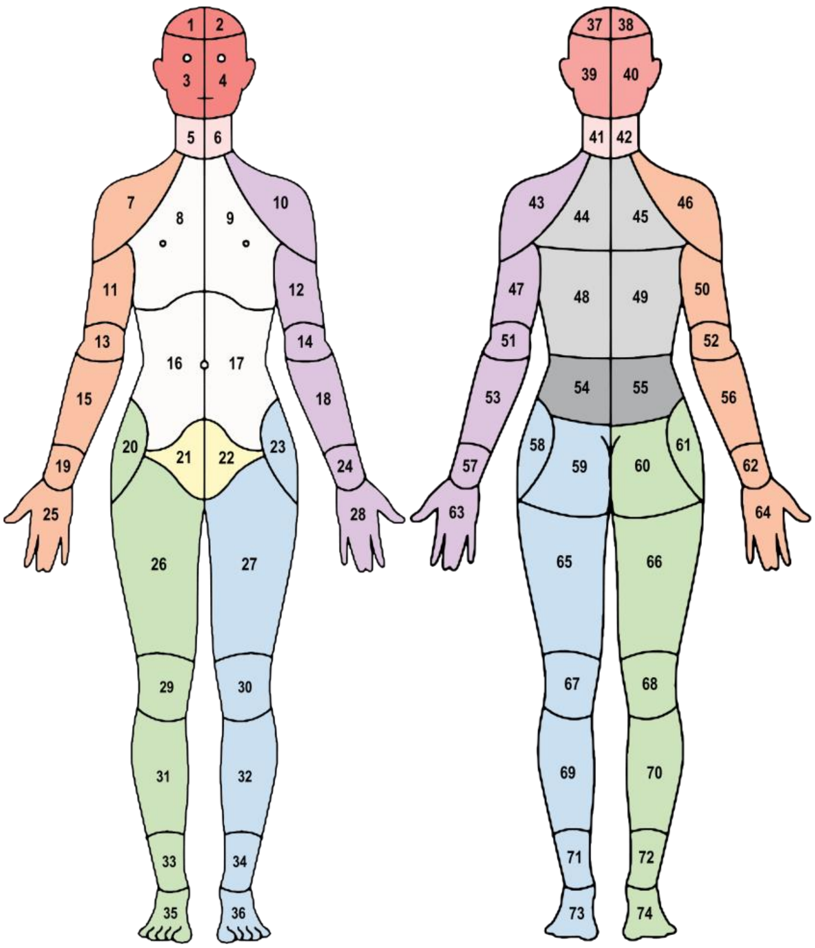

Fig. S1. CHOIR body map with 74 numbered segments and 11 colored body regions (see also Table S2).

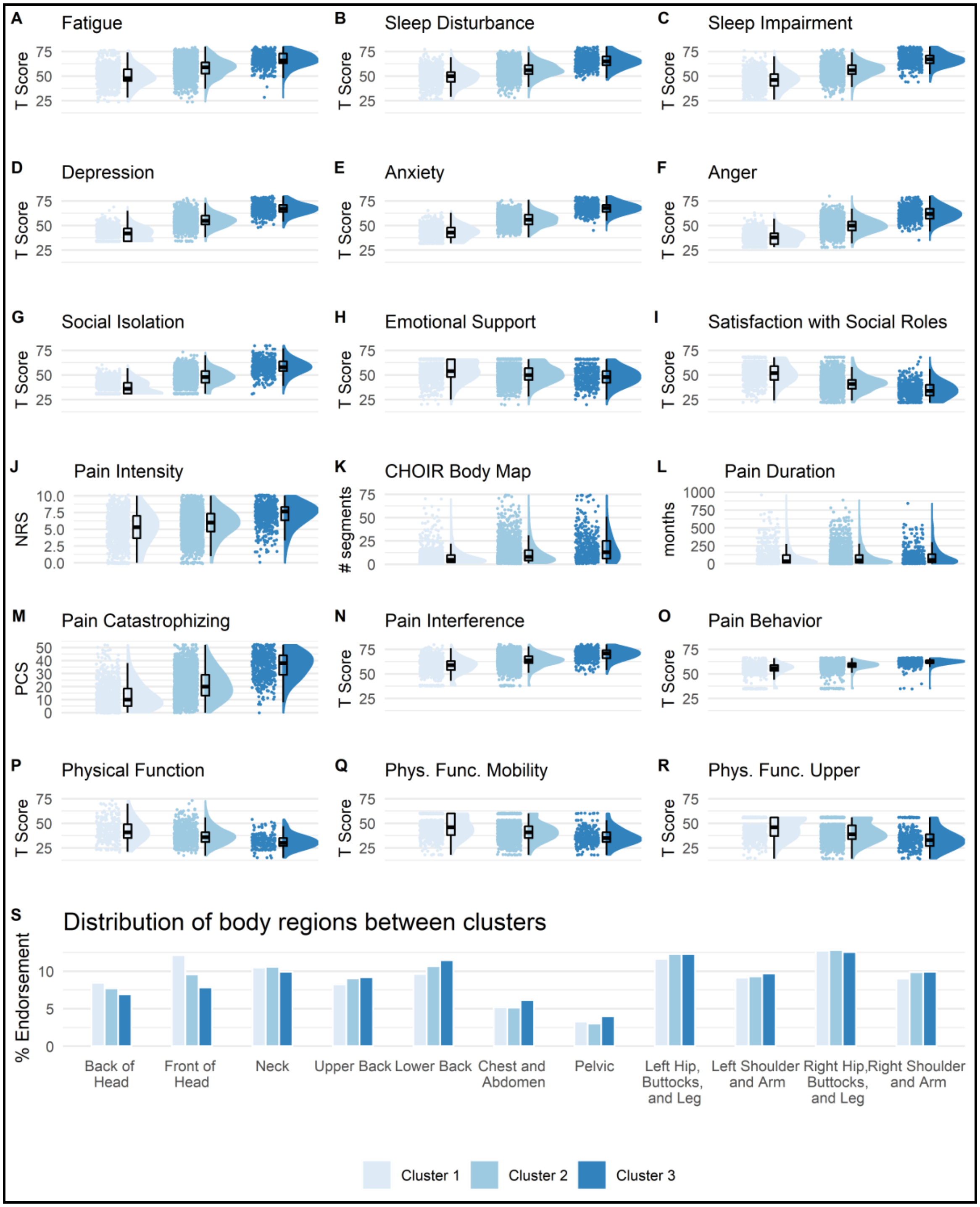

Fig. S2. Clusters validation in the *validation* dataset (n=3817). A graded scale of severity is manifested again across all clustering symptoms (A-I), as well as on almost all pain-specific measures (J-R), except for pain duration (L, *p*=0.724), such that Cluster1 reflects a low severity, Cluster2 a medium severity, and Cluster3 the worst severity. Raincloud plots combining jittered raw data, data distribution, and boxplots were generated using open source code (*93*). Complementary descriptive and inferential statistical information is provided in Table S3. (S) The plot shows the % endorsement of 11 body regions as distributed in each of the clusters. There was no significant association in the distribution of % endorsed body regions between the clusters (Chi^2=1.71, *p*=0.99). NRS=Numerical Rating Scale; PCS=Pain Catastrophizing Scale.

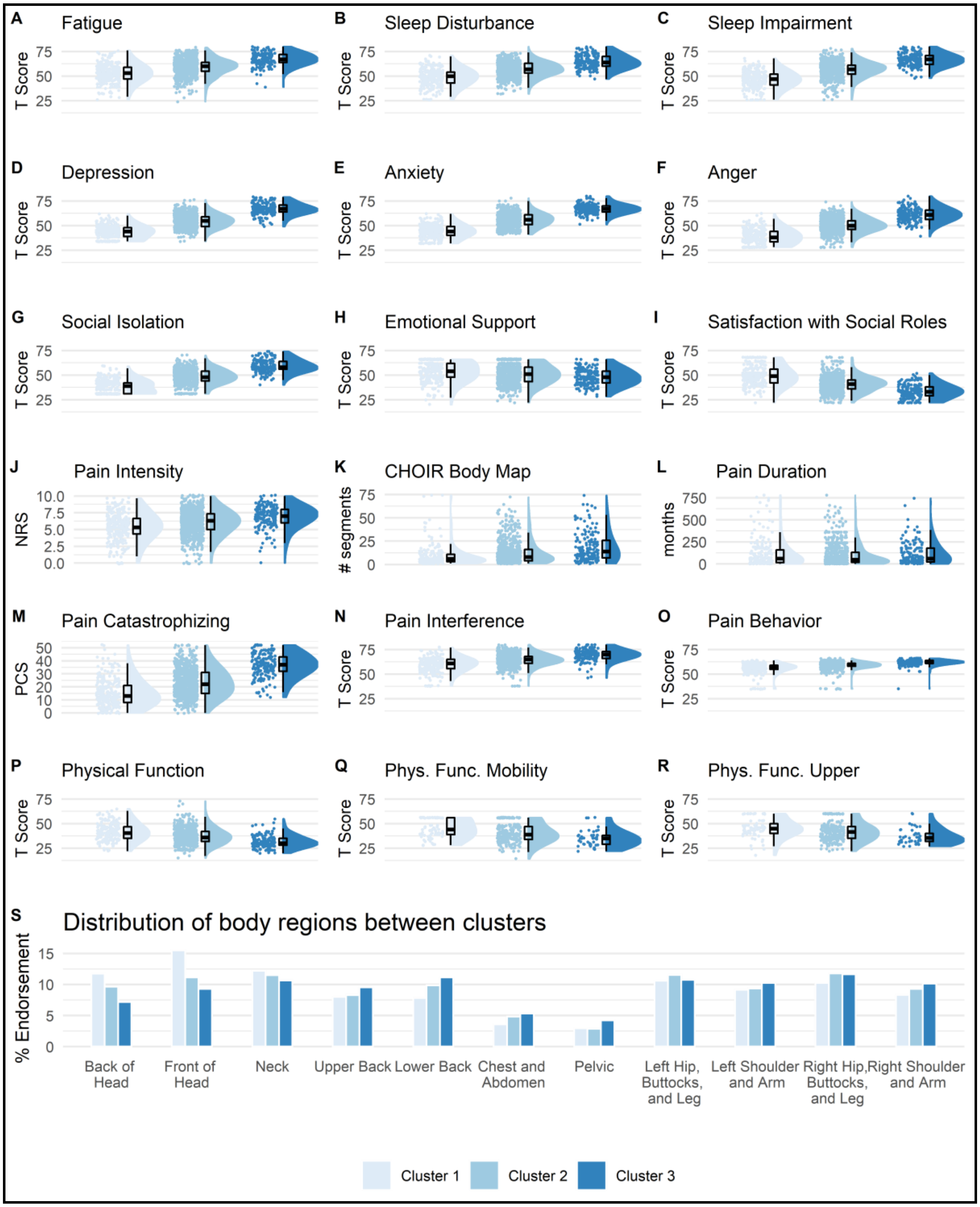

Fig. S3. Clusters validation in the *longitudinal* dataset, at baseline (n=1273). A graded scale of severity is manifested again across all clustering symptoms (A-I), as well as on almost all pain-specific measures (J-R), except for pain duration (L, p=0.12), such that Cluster1 reflects a low severity, Cluster2 a medium severity, and Cluster3 the worst severity. Raincloud plots combining jittered raw data, data distribution, and boxplots were generated using open source code (*93*). Complementary descriptive and inferential statistical information is provided in Table S4. (S) The plot shows the % endorsement of 11 body regions as distributed in each of the clusters. There was no significant association in the distribution of % endorsed body regions between the clusters (Chi^2=4.75, p=0.99). NRS=Numerical Rating Scale; PCS=Pain Catastrophizing Scale.

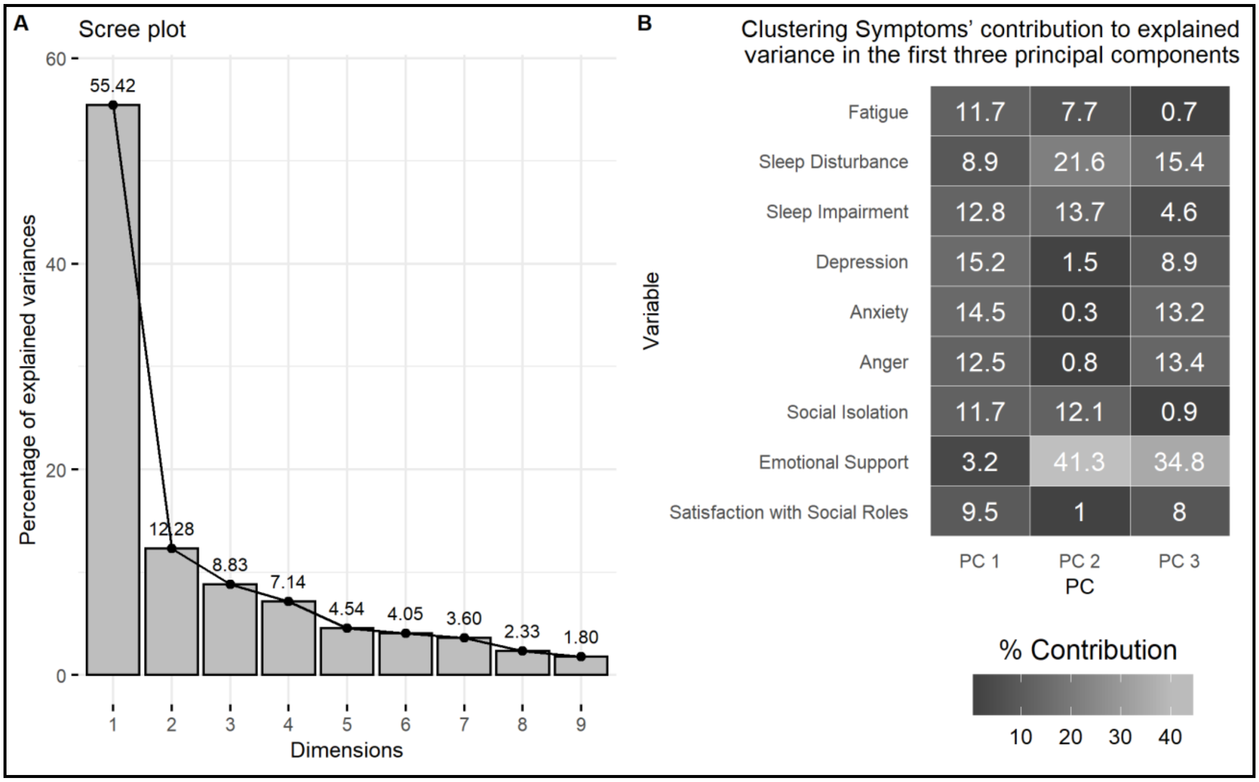

Fig. S4. Principal Component Analysis (PCA) applied on the nine clustering symptoms. (**A**) Scree plot indicating the percent of explained variance in the data by each of the nine principal components (PCs). For example, 55.42% of the variance is explained by PC 1. (**B**) The percent contribution of each of the nine clustering symptoms in accounting for the variability in the first three PCs. Variables that are mostly contributing to PC 1 are the most important in explaining the variability in the dataset. The negative affect-related factors Depression, Anxiety, and Anger, are ranked 1st, 2nd, and 4th, respectively, and contribute together 42.2% to PC 1.

N (%)	Total	Cluster1	Cluster2	Cluster3	p*
**Total:**	16538	4137 (25.02)	8975 (54.27)	3426 (20.72)	
**Age (years):**					0.97
18-29	2057 (12.44)	525 (12.69)	1070 (11.92)	462 (13.49)	
30-39	2794 (16.89)	701 (16.94)	1463 (16.3)	630 (18.39)	
40-49	3103 (18.76)	692 (16.73)	1640 (18.27)	771 (22.5)	
50-59	3497 (21.15)	776 (18.76)	1930 (21.5)	791 (23.09)	
60-69	2882 (17.43)	797 (19.27)	1606 (17.89)	479 (13.98)	
≥70	2173 (13.14)	638 (15.42)	1247 (13.89)	288 (8.41)	
No response	32 (0.19)	8 (0.19)	19 (0.21)	5 (0.15)	
**Sex**					0.96
Female	10701 (64.71)	2578 (62.32)	5824 (64.89)	2299 (67.1)	
Male	5283 (31.94)	1431 (34.59)	2841 (31.65)	1011 (29.51)	
No response	554 (3.35)	128 (3.09)	310 (3.45)	116 (3.39)	
**Ethnicity**					0.97
Hispanic/Latino	1677 (10.14)	429 (10.37)	807 (8.99)	441 (12.87)	
Non-Hispanic/Non-Latino	12322 (74.51)	3125 (75.54)	6800 (75.77)	2397 (69.96)	
Patient refused	514 (3.11)	137 (3.31)	278 (3.1)	99 (2.89)	
Unknown	611 (3.69)	168 (4.06)	331 (3.69)	112 (3.27)	
No response	1414 (8.55)	278 (6.72)	759 (8.46)	377 (11)	
**Race**					0.99
American Indian or Alaska Native	79 (0.48)	17 (0.41)	40 (0.45)	22 (0.64)	
Asian	1348 (8.15)	397 (9.6)	714 (7.96)	237 (6.92)	
Asian, non-Hispanic	10 (0.06)	1 (0.02)	6 (0.07)	3 (0.09)	
Black or African American	560 (3.39)	141 (3.41)	270 (3.01)	149 (4.35)	
Black, non-Hispanic	10 (0.06)	4 (0.1)	3 (0.03)	3 (0.09)	
Native American, Hispanic	1 (0.01)	0 (0)	0 (0)	1 (0.03)	
Native American, non-Hispanic	2 (0.01)	1 (0.02)	1 (0.01)	0 (0)	
Native Hawaiian or Other Pacific	83 (0.5)	19 (0.46)	44 (0.49)	20 (0.58)	
Other	2807 (16.97)	706 (17.07)	1453 (16.19)	648 (18.91)	
Other, Hispanic	16 (0.1)	3 (0.07)	12 (0.13)	1 (0.03)	
Other, non-Hispanic	8 (0.05)	2 (0.05)	5 (0.06)	1 (0.03)	
Patient Refused	482 (2.91)	128 (3.09)	258 (2.87)	96 (2.8)	
Unknown	628 (3.8)	163 (3.94)	342 (3.81)	123 (3.59)	
White	8909 (53.87)	2226 (53.81)	4970 (55.38)	1713 (50)	
White, Hispanic	3 (0.02)	1 (0.02)	1 (0.01)	1 (0.03)	
White, non-Hispanic	154 (0.93)	41 (0.99)	86 (0.96)	27 (0.79)	
No response	1438 (8.7)	287 (6.94)	770 (8.58)	381 (11.12)	
**Marital Status**					0.72
Married	8648 (52.29)	2489 (60.16)	4722 (52.61)	1437 (41.91)	
Separated	355 (2.15)	54 (1.31)	182 (2.03)	119 (3.47)	
Widowed	636 (3.85)	162 (3.92)	357 (3.98)	117 (3.42)	
Never Married	3105 (18.77)	700 (16.92)	1659 (18.48)	746 (21.77)	
Living Together	994 (6.01)	230 (5.56)	546 (6.08)	218 (6.36)	
Divorced	1771 (10.71)	337 (8.15)	973 (10.84)	461 (13.46)	
No response	1029 (6.22)	165 (3.99)	536 (5.97)	328 (9.57)	
**Education (years):**					0.36
≤12	471 (2.85)	102 (2.47)	224 (2.5)	145 (4.23)	
13-16	4870 (29.45)	1055 (25.5)	2537 (28.27)	1278 (37.3)	
17-20	8775 (53.06)	2375 (57.41)	4882 (54.4)	1518 (44.31)	
≥21	1465 (8.86)	443 (10.71)	838 (9.34)	184 (5.37)	
No response	957 (5.79)	162 (3.92)	494 (5.5)	301 (8.79)	

Table S1. Participants' demographic information for the entire dataset and across the three clusters. Number of patients is indicated, with % in parenthesis. * Reflects the results of a Chi^2

test (categories with less than a minimum of 5 patients per group were removed) comparing across clusters.

Body region	CHOIR body map segments
Front of Head	1, 2, 3, 4
Back of Head	37, 38, 39, 40
Neck	5, 6, 41, 42
Chest/Abdomen	8, 9, 16, 17
Upper Back	44, 45, 48, 49
Lower Back	54, 55
Pelvic	21, 22
Right Shoulder/Arm	7, 11, 13, 15, 19, 25, 46, 50, 52, 56, 62, 64
Left Shoulder/Arm	10, 12, 14, 18, 24, 28, 43, 47, 51, 53, 57, 63
Right Hip/Buttocks/Leg	20, 26, 29, 31, 33, 35, 60, 61, 66, 68, 70, 72, 74
Left Hip/Buttocks/Leg	23, 27, 30, 32, 34, 36, 58, 59, 65, 67, 69, 71, 73

Table S2. CHOIR body map regions and their associated segments (see also Fig. S1).

	Descriptive (M±SD)			Main Effect of Cluster			C1 vs. C2		C1 vs. C3		C2 vs. C3	
	C1	C2	C3	F	df	*P**	*P^*	Cohen's D	*P^*	Cohen's D	*P^*	Cohen's D
**Clustering symptoms:**												
Fatigue	49.84±9.68	58.35±8.96	67.37±7.61	676.62	2, 3814	<0.0001	<0.0001	0.91	<0.0001	2.01	<0.0001	1.09
Sleep Disturbance	49.13±8.67	56.45±7.29	65.97±7.54	830.78	2, 3814	<0.0001	<0.0001	0.91	<0.0001	2.07	<0.0001	1.28
Sleep Impairment	46.13±9.1	56.56±7.28	67.33±6.86	1344.73	2, 3814	<0.0001	<0.0001	1.27	<0.0001	2.63	<0.0001	1.52
Depression	41.77±6.53	54.84±6.59	67.23±5.93	2785.15	2, 3814	<0.0001	<0.0001	1.99	<0.0001	4.08	<0.0001	1.98
Anxiety	43.14±6.75	55.98±6.37	68.12±5.68	2773.84	2, 3814	<0.0001	<0.0001	1.96	<0.0001	4.00	<0.0001	2.01
Anger	37.58±6.96	50.05±7.03	62.33±7.65	2175.22	2, 3814	<0.0001	<0.0001	1.78	<0.0001	3.38	<0.0001	1.67
Social Isolation	37.25±6.65	47.94±7.36	58.16±7.57	1511.44	2, 3814	<0.0001	<0.0001	1.52	<0.0001	2.93	<0.0001	1.37
Emotional Support	53.99±10.11	51.04±8.98	48.68±8.84	61.47	2, 3814	<0.0001	<0.0001	0.31	<0.0001	0.56	<0.0001	0.26
Satisfaction w/ Soc. Roles	51.93±10.04	41.79±8.17	34.73±8.14	762.22	2, 3814	<0.0001	<0.0001	1.11	<0.0001	1.88	<0.0001	0.87
**Pain-specific measures:**												
Pain Intensity	5.13±2.43	5.89±1.96	7.21±1.79	173.39	2, 3814	<0.0001	<0.0001	0.40	<0.0001	1.50	<0.0001	1.04
# Bodymap Segments	8.46±9.68	11.69±11.39	17.71±15.67	88.49	2, 3279	<0.0001	<0.0001	0.24	<0.0001	1.15	<0.0001	0.54
Pain Duration	95.14±132.12	97.03±124.25	99.51±119.86	0.17	2, 3613	0.846	0.724	0.02	0.575	0.05	0.71	0.03
Pain Interference	58±8.34	63.79±6.56	70.39±5.87	557.71	2, 3814	<0.0001	<0.0001	0.93	<0.0001	2.67	<0.0001	1.59
Pain Behavior	54.89±6.73	58.65±4.38	61.92±3.32	371.14	2, 3814	<0.0001	<0.0001	0.97	<0.0001	2.27	<0.0001	1.39
Physical function	42.62±10.71	37.26±8.49	31.44±6.92	106.97	2, 1378	<0.0001	<0.0001	0.69	<0.0001	1.86	<0.0001	1.19
Phys. Func.-Mobility	46.09±10.63	41.75±9.49	36.17±8.42	111.29	2, 2433	<0.0001	<0.0001	0.48	<0.0001	1.48	<0.0001	0.94
Phys. Func.-Up. Extrem.	45.28±10.28	40.79±10.05	34±10.23	128.48	2, 2433	<0.0001	<0.0001	0.44	<0.0001	1.59	<0.0001	0.94
Pain Catastrophizing	12.86±10.19	21.6±11.12	36.37±9.91	753.77	2, 3598	<0.0001	<0.0001	0.83	<0.0001	2.99	<0.0001	2.11

Table S3. Clustering symptoms and pain-specific measures as per the three clusters in the *validation* dataset (n=3817). M=mean, SD=standard deviation; C1=Cluster1, C2=Cluster2; C3=Cluster3. *Bonferroni threshold for ANOVA main effects of cluster is set at p=0.0028 ^Bonferroni threshold for t-test comparisons between each two clusters is set at p=0.0009

	Descriptive (M±SD)			Main Effect of Cluster			C1 vs. C2		C1 vs. C3		C2 vs. C3	
	C1	C2	C3	F	df	*P**	*P^*	Cohen's D	*P^*	Cohen's D	*P^*	Cohen's D
**Clustering symptoms:**												
Fatigue	53.02±9.47	59.37±8.6	67.62±7.16	155.85	2, 1270	<0.0001	<0.0001	0.70	<0.0001	1.74	<0.0001	1.04
Sleep Disturbance	48.68±8.87	56.85±7.23	64.93±7.2	252.70	2, 1270	<0.0001	<0.0001	1.01	<0.0001	2.01	<0.0001	1.12
Sleep Impairment	46.29±8.44	56.73±7.85	66.78±6.56	381.60	2, 1270	<0.0001	<0.0001	1.28	<0.0001	2.71	<0.0001	1.39
Depression	43.3±6.53	54.84±6.65	66.97±5.94	721.08	2, 1270	<0.0001	<0.0001	1.75	<0.0001	3.79	<0.0001	1.92
Anxiety	44.26±6.5	55.92±6.52	67.4±5.48	727.29	2, 1270	<0.0001	<0.0001	1.79	<0.0001	3.85	<0.0001	1.91
Anger	38.79±7.66	50.44±7.26	61.28±7.13	523.95	2, 1270	<0.0001	<0.0001	1.56	<0.0001	3.04	<0.0001	1.51
Social Isolation	38.24±6.84	48.5±7.13	59.19±6.44	495.58	2, 1270	<0.0001	<0.0001	1.47	<0.0001	3.15	<0.0001	1.57
Emotional Support	53.43±9.96	50.97±9.47	47.74±8.17	19.79	2, 1270	<0.0001	<0.0005	0.25	<0.0001	0.62	<0.0001	0.37
Satisfaction w/ Soc. Roles	49.6±9.91	41.02±7.81	33.52±6.81	218.01	2, 1270	<0.0001	<0.0001	0.96	<0.0001	1.89	<0.0001	1.02
**Pain-specific measures:**												
Pain Intensity	5.39±2.06	6.05±1.85	6.95±1.64	37.59	2, 1270	<0.0001	<0.0001	0.38	<0.0001	1.19	<0.0001	0.78
# Bodymap Segments	9.27±11.63	12.77±12.44	18.88±16.03	24.12	2, 980	<0.0001	0.0005	0.24	<0.0001	1.09	<0.0001	0.54
Pain Duration	118.85±161.08	99.14±123.74	114.22±128.99	2.10	2, 1017	0.12	0.06	0.16	0.78	0.05	0.19	0.17
Pain Interference	59.9±7.02	64.73±5.97	69.81±5.92	140.15	2, 1270	<0.0001	<0.0001	0.81	<0.0001	2.35	<0.0001	1.21
Pain Behavior	55.86±5.48	59.09±3.61	61.83±3.07	125.90	2, 1270	<0.0001	<0.0001	0.96	<0.0001	2.34	<0.0001	1.26
Physical function	41.42±8.82	37.23±8.16	31.74±6.33	58.40	2, 931	<0.0001	<0.0001	0.57	<0.0001	1.68	<0.0001	1.23
Phys. Func.-Mobility	45.1±9.1	40.41±9.53	34.98±8.36	16.21	2, 336	<0.0001	<0.0005	0.52	<0.0001	1.50	<0.001	0.92
Phys. Func.-Up. Extrem.	45.4±9.53	41.84±9.25	37.12±8.08	10.98	2, 336	<0.0001	<0.005	0.41	<0.0001	1.27	<0.005	0.83
Pain Catastrophizing	15.27±10.53	23.24±10.68	36.58±8.85	227.25	2, 1270	<0.0001	<0.0001	0.81	<0.0001	2.82	<0.0001	2.13

Table S4. Clustering symptoms and pain-specific measures as per the three clusters in the *longitudinal* dataset, at baseline (n=1273). M=mean, SD=standard deviation; C1=Cluster1, C2=Cluster2; C3=Cluster3. *Bonferroni threshold for ANOVA main effects of cluster is set at p=0.0028 ^Bonferroni threshold for t-test comparisons between each two clusters is set at p=0.0009

**Linkage methods**	**Agglomerative coefficient**
Single	0.440
Average	0.670
McQuitty	0.749
Complete	0.846
**Ward**	**0.920**

Table S5. Agglomerative coefficient values of various linkage methods that were combined with the Euclidian distance metric.
